# Salicylic acid‐dependent immunity contributes to resistance against *Rhizoctonia solani*, a necrotrophic fungal agent of sheath blight, in rice and *Brachypodium distachyon*

**DOI:** 10.1111/nph.14849

**Published:** 2017-10-19

**Authors:** Yusuke Kouzai, Mamiko Kimura, Megumi Watanabe, Kazuki Kusunoki, Daiki Osaka, Tomoko Suzuki, Hidenori Matsui, Mikihiro Yamamoto, Yuki Ichinose, Kazuhiro Toyoda, Takakazu Matsuura, Izumi C. Mori, Takashi Hirayama, Eiichi Minami, Yoko Nishizawa, Komaki Inoue, Yoshihiko Onda, Keiichi Mochida, Yoshiteru Noutoshi

**Affiliations:** ^1^ Graduate School of Environmental and Life Science Okayama University Kita‐ku Okayama 700‐8530 Japan; ^2^ Cellulose Production Research Team Biomass Engineering Research Division RIKEN Center for Sustainable Resource Science Tsurumi Yokohama 230‐0045 Japan; ^3^ Department of Science Japan Women's University, Mejirodai Bunkyo‐ku Tokyo 112‐8681 Japan; ^4^ Institute of Plant Science and Resources (IPSR) Okayama University Kurashiki 710‐0046 Japan; ^5^ Division of Plant and Microbial Sciences Institute of Agrobiological Sciences National Agriculture and Food Research Organization (NARO) Tsukuba 305‐8602 Japan; ^6^ Kihara Institute for Biological Research Yokohama City University 641‐12 Maioka‐cho, Totsuka‐ku Yokohama 244‐0813 Japan

**Keywords:** biotroph, *Brachypodium distachyon*, disease resistance, necrotroph, *Rhizoctonia solani*, rice, salicylic acid (SA), sheath blight

## Abstract

- *Rhizoctonia solani* is a soil‐borne fungus causing sheath blight. In consistent with its necrotrophic life style, no rice cultivars fully resistant to *R. solani* are known, and agrochemical plant defense activators used for rice blast, which upregulate a phytohormonal salicylic acid (SA)‐dependent pathway, are ineffective towards this pathogen. As a result of the unavailability of genetics, the infection process of *R. solani* remains unclear.
- We used the model monocotyledonous plants *Brachypodium distachyon* and rice, and evaluated the effects of phytohormone‐induced resistance to *R. solani* by pharmacological, genetic and microscopic approaches to understand fungal pathogenicity.
- Pretreatment with SA, but not with plant defense activators used in agriculture, can unexpectedly induce sheath blight resistance in plants. SA treatment inhibits the advancement of *R. solani* to the point in the infection process in which fungal biomass shows remarkable expansion and specific infection machinery is developed. The involvement of SA in *R. solani* resistance is demonstrated by SA‐deficient *NahG* transgenic rice and the sheath blight‐resistant *B. distachyon* accessions, Bd3‐1 and Gaz‐4, which activate SA‐dependent signaling on inoculation.
- Our findings suggest a hemi‐biotrophic nature of *R. solani*, which can be targeted by SA‐dependent plant immunity. Furthermore, *B. distachyon* provides a genetic resource that can confer disease resistance against *R. solani* to plants.

## Introduction

Sheath blight, caused by the soil‐borne fungal phytopathogen *Rhizoctonia solani* Kühn, is a major disease of cultivated rice (*Oryza sativa*). It causes severe agricultural and economic losses, especially in East Asia and southern USA (Lee & Rush, 1983). In the paddy field, the infection process of *R. solani* is initiated by the attachment of sclerotia, masses of compacted mycelium that can survive under unfavorable environmental conditions, to the leaf sheath of rice. Oval necrotic lesions are formed on the sheath near the water line, and the infection extends to the upper parts of the plant. The aerial hyphae from the affected plants infect adjacent plants in the field, and growth defects, lodging and death occur, leading to yield losses. In *c*. 1350 rice cultivars, none was found to be completely resistant to sheath blight, although the level of disease symptoms varied (Hashiba, 1984). Thus, at present, sheath blight disease is managed using fungicides in the field. However, quinone outside inhibitor (QoI) fungicides were unable to control *R. solani* in the USA in 2011 owing to the development of resistance isolates (Olaya *et al*., 2012). In addition, as warm temperature and high humidity are favorable for the growth of *R. solani*, global warming could further increase the future risk of agricultural damage by this pathogen.

Plant pathogens are divided into biotrophs and necrotrophs on the basis of their nutrient feeding strategies (Glazebrook, 2005). Obligate biotrophs infect specific plant species and draw sustenance from living host tissues. Necrotrophs kill host cells, often by producing phytotoxins, and feed on damaged tissues. Pathogens for which the infection process involves both biotrophic and necrotrophic phases are called hemi‐biotrophs. Most such pathogens initially infect hosts biotrophically and then shift to the necrotrophic stage. The transition time depends on the pathogen type. Based on the disease symptoms with severe necrotic lesions and its broad host range, *R. solani* is considered to be a simple necrotrophic parasite. Indeed, *R. solani* is also known to infect > 200 crop species and induces seed decay, seedling damping‐off, stem canker, black scurf and root rot.

Plants use a phytohormone, salicylic acid (SA), to induce defense responses against biotrophic pathogens. The SA‐dependent signaling pathway leads to the production of antimicrobial molecules, such as pathogenesis‐related proteins and phytoalexins, as well as the induction of hypersensitive responses (HR), often associated with programmed cell death in the infected area, which restricts pathogen colonization. By contrast, plants use the phytohormones jasmonic acid (JA) and/or ethylene (ET) for defense against necrotrophs, and their responses are similar to those of wounding. Exogenously applied JA or ET suppressed the disease symptoms caused by infection with the necrotrophic fungal pathogens *Botrytis cinerea* and *Alternaria brassicicola* in *Arabidopsis thaliana* (Thomma *et al*., 1999). The application of SA to *A. thaliana* decreased resistance to *A. brassicicola*, probably as a result of its antagonistic effect to JA (Spoel *et al*., 2007). By contrast, SA increased *A. thaliana* resistance to *B. cinerea*, and the importance of endogenous SA, probably through phenylalanine ammonia lyase, in the resistance to *B. cinerea* has been demonstrated (Ferrari *et al*., 2003). The effectiveness of phytohormone application on induced resistance is dependent on the pathogen's infection strategy. For the control of rice blast caused by a hemi‐biotrophic pathogen, *Pyricularia oryzae*, agrochemical immune potentiators, called plant defense activators, have been used successfully. Agrochemicals such as probenazole, tiadinil and isotianil boost the SA‐related defense response in plants, thereby conferring resistance, even to virulent pathogens. As such chemicals target host plants, no drug‐resistant pathogens have yet been identified. Therefore, they provide durable and sustainable crop protection. However, the plant defense activators used in agriculture have no crop protection effects against *R. solani*. The increase in plant resistance to sheath blight disease has been investigated using genetic engineering approaches to fortify plant responses to JA or ET. Transgenic rice with enhanced production of pathogen‐induced ET was found to be more resistant to *R. solani* (Helliwell *et al*., 2013). The overexpression of the *OsWRKY30* gene in rice induced an elevated level of JA accumulation after infection and increased resistance to *R. solani* (Peng *et al*., 2012).

The entire genome sequence of *R. solani* has been revealed and several secreted proteins have been identified as potential agents for necrosis in *Arabidopsis* (Zheng *et al*., 2013). However, the virulence mechanism of *R. solani* remains unclear, as genetic approaches cannot be applied owing to its multinucleate and heterokaryotic nature. The pathogen's pathogenicity and the plant's defense system at the molecular level can be determined using an appropriate model pathosystem. Brachypoideae belongs to a tribe which configures the core Pooideae with Littledaleeae, Bromeae and Triticeae (Soreng *et al*., 2015), and a small grass, *B. distachyon*, is used as an experimental model plant for economically important cereals such as wheat, barley and rice because of its short life cycle, self‐fertility and small diploid genome (Kellogg, 2015). The complete genome sequence and cDNA database of the standard accession Bd21 and a number of accessions are available (Vogel *et al*., 2006, 2009, 2010; Mochida *et al*., 2013). Various major crop pathogens can infect *B. distachyon* (Sandoya & Buanafina, 2014; Fitzgerald *et al*., 2015). *Brachypodium distachyon* has been used as a model for the investigation of wheat root rot disease caused by *R. solani* AG‐8 (Schneebeli *et al*., 2016).

In this study, we used *B. distachyon* and rice as hosts to investigate sheath blight disease and, unexpectedly, found that exogenously applied SA can induce sheath blight resistance in plants. The decreased level of endogenous SA induced by the transformed bacterial salicylate hydroxylase gene makes rice more susceptible to *R. solani*. We also identified two *R. solani*‐resistant accessions of *B. distachyon*, in which SA signaling was rapidly activated in response to fungal inoculation, indicating the existence of disease resistance genes for *R. solani*. The protective effect of plants against sheath blight disease by chemically and genetically induced SA‐dependent immunity suggests a two‐phase infection process of *R. solani*, with a preceding biotrophic stage and a subsequent necrotrophic stage as a novel aspect of its infection strategy. Our study demonstrates the possibility of the application of durable and sustainable crop protection methods, such as disease resistance genes or chemical defense activators, to *Rhizoctonia* diseases.

## Materials and Methods

### Plant and fungal materials

The *B. distachyon* accessions Bd21, Bd3‐1 and Gaz‐4 were obtained from the National Plant Germplasm System of the United States Department of Agriculture‐Agricultural Research Service (USDA‐ARS) (Vogel *et al*., 2006, 2009). Dry seeds were germinated on moist filter paper in a plastic Petri dish. After 7 d, the seedlings were transferred to wells of 24‐well microplates filled with soil (Sakata Supermix‐A; Sakata Seed, Yokohama, Kanagawa, Japan) and grown for 3–4 wk in a growth chamber with LED lights (Nippon Medical & Chemical Instruments, Osaka, Japan) at 23°C under a 20 h : 4 h, light : dark photoperiod. The 12 *R. solani* isolates and *B. cinerea* (MAFF237696) were provided by Genebank of the National Agricultural Research Organization (NARO) in Japan, and *P. oryzae* Guy11 was kindly provided by Dr Yukio Tosa and cultured on potato dextrose agar (PDA; BD, Franklin Lakes, NJ, USA) plates at 23°C for 3–5 d. Rice plants (*Oryza sativa* L. *japonica*) carrying the blast resistance genes *Pia* and *Pish* (cv Nipponbare (Pia)) were used as the wild‐type. Transgenic rice plants expressing *NahG* which had the ‘Nipponbare (Pia)’ background were kindly provided by Dr Chang‐Jie Jiang. Rice plants were grown hydroponically in nutrient solution under a photoperiod of 14 h of light (28°C) and 10 h of darkness (25°C).

### Phytohormones and chemical treatments

Sodium salicylate (Wako, Osaka, Japan), methyl jasmonate (Wako) and ethephon (Sigma‐Aldrich, St Louis, MO, USA), an ET generator, were used as phytohormones. Acetylsalicylic acid (Ac‐SA) (Nacalai Tesque, Kyoto, Japan), 3,5‐dichloroanthranilic acid (DCA) (Tokyo Chemical Industry, Tokyo, Japan), 2,6‐dichlorisonicotianic acid (INA) (Wako) and acibenzolar‐*S*‐methyl (benzothiadiazole, BTH) (Wako) were used as structural or functional analogs of SA. All phytohormones and chemicals were diluted with water or dimethyl sulfoxide (DMSO). The *B. distachyon* seedlings or detached leaves were sprayed, soaked or soil‐drenched with a chemical solution containing 0.04% (v/v) Tween 20 and incubated for 24 or 48 h at 23°C. Droplets of chemical solution adhering to the leaf surface were wiped off before the following experiments.

### Inoculation tests

Detached shoots of *B. distachyon* were used for the screening of pathogenic *R. solani* strains, and detached leaves of *B. distachyon* and rice were used for other inoculation tests. Detached plants were placed on moist filter paper in a Petri dish for *B. distachyon* or a square plate for rice. The detached site was covered with moist Kimwipes (Nippon Paper Crecia, Tokyo, Japan). After 1 d of wound acclimation, cubic mycelial plugs (2–3 mm) cut from the edge of *R. solani* mycelia growing on PDA plates were placed on the leaf sheath or in the middle of detached leaves as inoculum. The lid of the plate was closed and sealed immediately with surgical tape to avoid drying. The plates were incubated at 23°C and 25°C for *B. distachyon* and rice, respectively, under continuous light. For the inoculation of *P. oryzae* and *B. cinerea*, conidial suspensions were spotted onto leaves. To quantify disease severity, the fungal biomass in the inoculated leaves was measured by qPCR according to previous reports (Sayler & Yang, 2007; Budge *et al*., 2009). Surface fungal mycelia of the inoculated leaves were removed by wet‐wipes with 70% ethanol and adhesive tape, and DNA was extracted using a Nucleospin Plant II Kit (Takara Bio, Shiga, Japan). PCR for *R. solani* DNAs (28S rDNA for MAFF305230, a tubulin gene for MAFF305256) was performed using a KAPA SYBR Fast qPCR Kit (Kapa Biosystems, Woburn, MA, USA) with a GVP‐9600 instrument (Shimadzu, Kyoto, Japan) or SYBR Premix Ex Taq II (Takara Bio) with an Applied Biosystems 7500 System (Thermo Fisher Scientific, Waltham, MA, USA). The *B. distachyon BdFIM* gene was used for normalization (Zhu *et al*., 2014). Primers are listed in Supporting Information Table S1.

### Microscopic observations

*Rhizoctonia solani* hyphae were stained with trypan blue (Wako) dissolved in 40 ml of 1 : 1 : 1 : 1 phenol/lactic acid/glycerol/water. The inoculated leaves were collected in 2‐ml tubes and boiled for 5 min in 1 : 1 ethanol/staining solution, followed by incubation for 30 min at room temperature. The samples were then washed with water three times and stored in 30% glycerol before microscopic observation.

### Gene expression analysis

Total RNAs were extracted from the inoculated leaves using an RNeasy Plant Mini Kit (Qiagen, Hilden, Germany) with on‐column DNase treatment. cDNAs were synthesized using an Omniscript RT Kit (Qiagen). Gene expression analysis was performed by quantitative reverse transcription‐polymerase chain reaction (qRT‐PCR) using SYBR Premix Ex Taq II with an Applied Biosystems 7500 System, and the data were normalized with the *BdUbi4* gene (*Bradi3 g04730*). Primers are listed in Table S1.

### Transcriptome analysis

Total RNAs were extracted from *B. distachyon* leaves after 24 h of treatment with 0.5 mM SA, 0.5 mM BTH or 0.5% (v/v) DMSO using an RNeasy Plant Mini Kit (QIAGEN). The quality and quantity of total RNAs were checked busing a NanoDrop spectrophotometer (Thermo Fisher Scientific) and a 2100 Bioanalyzer (Agilent, Santa Clara, CA, USA). Libraries for RNA‐seq were constructed for three biological replicates using a Truseq RNA library preparation kit (Illumina, San Diego, CA, USA) according to the manufacturer's instructions, and the libraries were sequenced using a Hiseq4000 sequencer (Illumina). The dataset of the Illumina reads has been submitted to the DNA Data Bank of Japan (DDBJ) Sequence Read Archive under the accession number DRA005681. The Illumina reads were mapped to the *B. distachyon* reference genome Bdistachyon_314 retrieved from Phytozome using TopHat v2.1.1 (Kim *et al*., 2013) with Bowtie v2.2.6 as its mapping tool (Langmead & Salzberg, 2012). The number of mapped reads of each gene was counted using FeatureCounts and normalized by reads per million (RPM). Genes with a mean RPM in three biological replicates in the same treatment of ≥ 1 were defined as expressed genes. Differentially expressed genes (DEGs) in SA and BTH treatments compared with the DMSO treatment as the control condition were determined based on the read counts of expressed genes using edgeR (McCarthy *et al*., 2012). The genes with a false discovery rate (FDR) from Fisher's exact test of < 0.01 and a log_2_ transformed fold change of > 1 or < −1 were identified as DEGs for each of the SA and BTH treatments. The distribution of DEGs in the three treatments was depicted using an R package: VennDiagram.

### Gene ontology enrichment analysis

Gene ontology (GO) enrichment analysis for the DEGs was performed to infer functional properties in the transcriptome responding to each of the treatments. For the GO enrichment analysis, GO terms were assigned to *Brachypodium* genes based on the GO annotations of their putative homologs of Arabidopsis which showed highest similarities in a blastp search with a threshold e‐value of < 1e‐5. Significantly over‐ or less‐represented GO terms were identified using the agriGO web service (http://bioinfo.cau.edu.cn/agriGO/index.php) with a threshold of FDR < 0.05 from Fisher's exact test compared with the GO terms assigned to all of the expressed genes as the background. The GO terms enriched in each set of DEGs were summarized based on their semantic similarities and represented on a two‐dimensional semantic space by the REVIGO web service (http://revigo.irb.hr/).

### Quantification of phytohormones

Approximately 50 mg of leaf blades were used for each extraction. The content of multiple phytohormones was measured by liquid chromatography‐tandem mass spectrometry (LC‐MS/MS), as reported previously (Mikami *et al*., 2016).

### Statistical analysis

Significance between populations was determined using unpaired Student's *t*‐tests. Graphs throughout the article show the mean value and error bars show the standard error (SE).

## Results

### Surveillance of infectivity of *R. solani* isolates on *B. distachyon*

**Figure 1:**
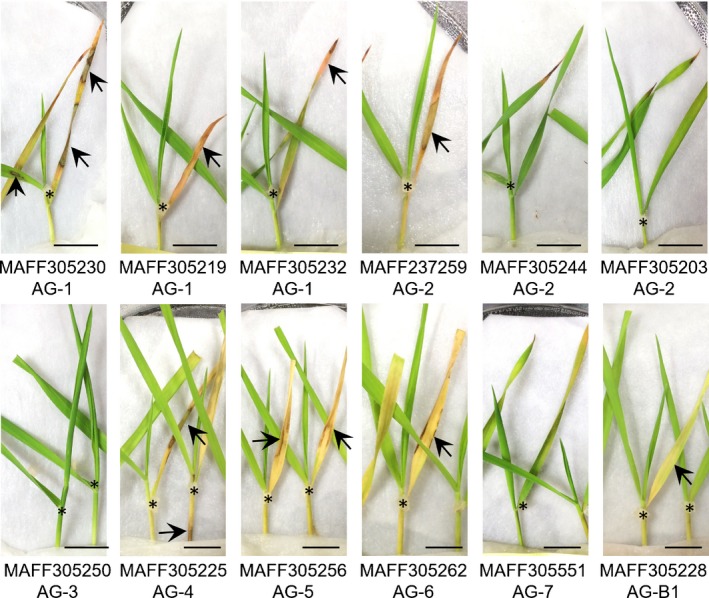
Infectivity of *Rhizoctonia solani* Japanese isolates on detached shoots of *Brachypodium distachyon* standard accession Bd21. Bd21 shoots were detached and inoculated with the different *R. solani* isolates listed in Table 1. Photographs were taken at 4 d post‐inoculation (dpi). Asterisks indicate the location of inoculum agar plugs, and arrows indicate necrotic lesions or etiolated regions of leaf blades, similar to symptoms typically observed in rice grown in the field. Bars, 1 cm. The assay was performed twice with similar results.

To analyze the molecular basis for both pathogen virulence and plant resistance in sheath blight disease, we used a model plant *B. distachyon*. We evaluated the infectivity of *R. solani* on *B. distachyon* standard accession Bd21 using 12 Japanese field isolates from various anastomosis groups (AGs) (Table 1), which were determined by hyphal fusion and were related to pathogenicity and host range (Anderson, 1982; Ogoshi, 1987). Mycelial plugs prepared from nutrient agar were inoculated on the detached shoots of *B. distachyon*. Eight fungal isolates formed visible lesions, and the leaf blades became etiolated by 2–3 d post‐inoculation (dpi) (Fig. 1). The AG‐1 IA isolate (MAFF305230), sampled from rice sheath blight which showed the most severe symptoms, was used for further analysis.

**Table 1:** Pathogenicity of *Rhizoctonia solani* Japanese isolates against *Brachypodium distachyon* Bd21

*R. solani* strains			Isolation source	Disease severity^b^
MAFF number^a^	Anastomosis group	Isolate name		
305230	AG‐1 IA	C‐325	Rice	**++**
305219	AG‐1 IA	C‐54	Rice	**+**
305232	AG‐1 IA	C‐501	Sudangrass	**+**
305203	AG‐2‐1 II	6	Six‐rowed barley	**–**
305244	AG‐2‐2 IIIB	C‐329	Rice	**–**
237259	AG‐2‐3	H5‐210	Wheat	**++**
305250	AG‐3 IV	C‐564	Potato	**–**
305225	AG‐4 IIIA	BO‐3	Cauliflower	**++**
305256	AG‐5	SH‐30	Soil	**++**
305262	AG‐6	UB‐7‐1‐A	Soil	**+**
305551	AG‐7	1529	Radish field soil	**–**
305228	AG‐BI	SH‐1‐2	Soil	**+**

a MAFF numbers are descriptors for the microorganism genetic resources assigned by the genetic resources center NARO (National Agriculture and Food Research Organization) in Japan (https://www.gene.affrc.go.jp/databases-micro_search_en.php).

b ++, severe symptoms; +, modest symptoms; –, no symptoms.

### Phytohormone‐induced resistance to *R. solani* in plants

**Figure 2:**
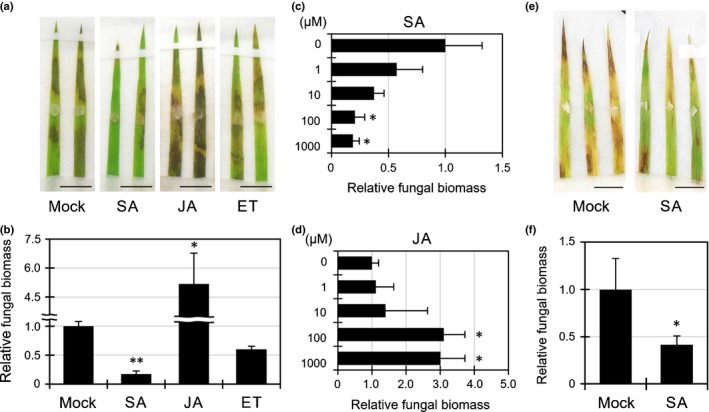
Salicylic acid (SA) induces *Rhizoctonia solani* resistance in *Brachypodium distachyon*. (a, b) Lesion formation (a) and relative biomass (linear scale) (b) of *R. solani* AG‐1 on *B. distachyon* leaves treated with water (Mock) or SA, jasmonic acid (JA) or ethylene (ET) (1 mM each) at 3 d post‐inoculation (dpi). Bars, 1 cm. Data are represented as means ± SEM,* n* = 4; *, *P* < 0.05; **, *P* < 0.01 using Student's *t*‐tests. (c, d) Relative biomass of *R. solani* AG‐1 in *B. distachyon* leaves treated with 0, 1, 10, 100 and 1000 μM of SA (c) or JA (d) at 3 dpi. Data are represented as means ± SEM,* n* = 3; *, *P* < 0.05 using Student's *t*‐tests. (e, f) Lesion formation (e) and relative biomass (linear scale) (f) of *R. solani* AG‐5 in *B. distachyon* leaves treated with water (Mock) or 1 mM SA at 3 dpi. Bars, 1 cm. Data are represented as means ± SEM,* n* = 4; *, *P* < 0.05 using Student's *t*‐tests. All experiments were repeated three times with similar results.

**Figure 3:**
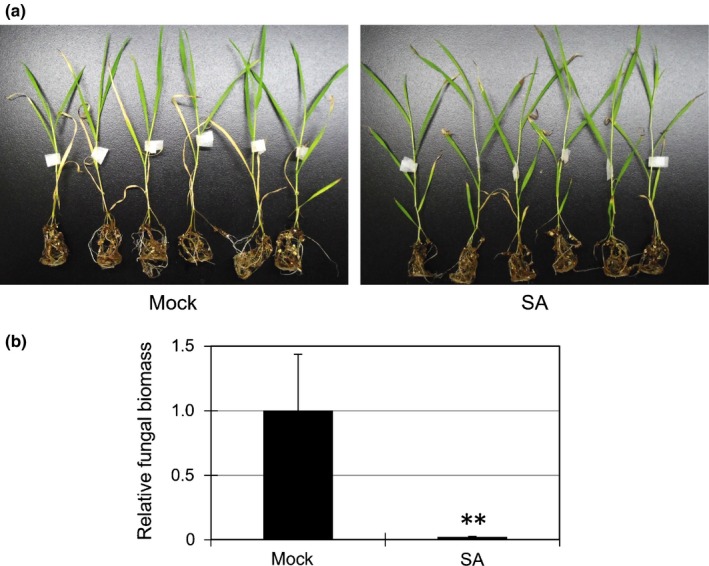
Salicylic acid (SA) induces *Rhizoctonia solani* resistance in soil‐grown *Brachypodium distachyon* intact plants. (a, b) Disease symptoms (a) and fungal biomass (linear scale) in leaves (b) of *R. solani* AG‐1 in *B. distachyon* intact plants grown on soil treated with water (Mock) or 1 mM SA. Bd21 whole plants grown on soil for 3 wk were sprayed with water (Mock) or 1 mM SA for 48 h and inoculated with *R. solani* (MAFF305230) by a mycelial agar plug held on the stem with tape. Photographs were taken at 7 d post‐inoculation (dpi). Data are represented as means ± SEM,* n* = 4; **, *P* < 0.01 using Student's *t*‐tests. Similar results were obtained in three independent experiments.

To test whether *B. distachyon* has the potential to induce resistance to *R. solani*, we treated Bd21 seedlings with SA, JA or ET, and inoculated seedlings with the pathogen. Contrary to our expectation, spray treatment of SA on detached leaf blades suppressed lesion formation and fungal biomass growth in *R. solani*‐inoculated leaves (Fig. 2a,b). By contrast, JA enhanced the susceptibility and ET had no effect on the symptoms. A concentration‐dependent analysis confirmed the effects of SA and JA on *R. solani* resistance (Fig. 2c,d). To further examine the effect of SA on *R. solani* resistance, we treated Bd21 intact plants with SA and inoculated them with the pathogen. Both the disease symptoms and *R. solani* foliar biomass were also suppressed by SA treatment in undetached *B. distachyon* (Fig. 3a,b). The phytohormones themselves did not inhibit fungal growth on agar medium (Fig. S1). To determine whether our experimental system could appropriately evaluate phytohormone‐induced plant defense, we inoculated a hemi‐biotrophic pathogen, *P. oryzae* strain Guy 11, and a necrotrophic pathogen, *Botrytis cinerea*, on *B. distachyon* (Govrin & Levine, 2002; Ebbole, 2007). In accord with the well‐known infection strategies of these fungi, treatments with SA and ET induced resistance to *P. oryzae* and *B. cinerea*, respectively (Fig. S2). SA application to *B. distachyon* also enhanced resistance against a different isolate of *R. solani* AG‐5 (MAFF305256) (Fig. 2e,f), indicating that SA‐induced immunity in *B. distachyon* is independent of, at least, these two fungal AGs.

**Figure 4:**
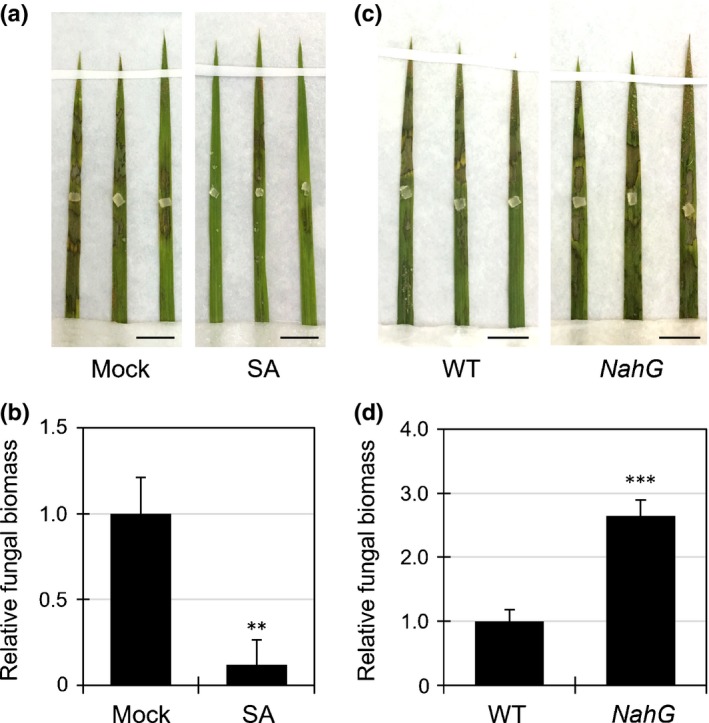
Salicylic acid (SA) induces and is required for *Rhizoctonia solani* resistance in rice. (a, b) Lesion formation (a) and fungal biomass (linear scale) (b) of *R. solani* AG‐1 in rice leaves treated with water (Mock) or 1 mM SA. Photographs were taken at 3 d post‐inoculation (dpi). The graph shows the relative *R. solani* biomass in the inoculated leaves at 3 dpi. Bars, 1 cm. Data are represented as means ± SEM of values relative to the mock treatment, *n* = 12; **, *P* < 0.01 using Student's *t*‐test. (c, d) Lesion formation (c) and relative biomass (linear scale) (d) of *R. solani* AG‐1 in the leaves of wild‐type and *NahG*‐overexpressing transgenic rice at 3 dpi. Bars, 1 cm. Data are represented as means ± SEM,* n *= 12; ***, *P *< 0.001 using Student's *t*‐tests. All experiments were repeated three times with similar results.

To determine whether these unexpected results were specific to *B. distachyon*, we conducted the same experiment using rice. Suppression of *R. solani* infection was also observed in SA‐treated rice, as evidenced by decreases in lesion formation and foliar fungal biomass (Fig. 4a,b).

### Contribution of SA to *R. solani* resistance in rice

To test whether innate SA signaling contributes to *R. solani* resistance in plants, we used transgenic rice plants expressing a bacterial SA hydroxylase (the *NahG* gene); this gene causes SA deficiency, leading to the loss of resistance to biotrophic pathogens (Yang *et al*., 2004). Rice *NahG* plants inoculated with *R. solani* were more susceptible than wild‐type plants (Fig. 4c,d). These results indicate that SA signaling plays a positive role in plant resistance to *R. solani*.

### Infection process of *R. solani* on *B. distachyon*

**Figure 5:**
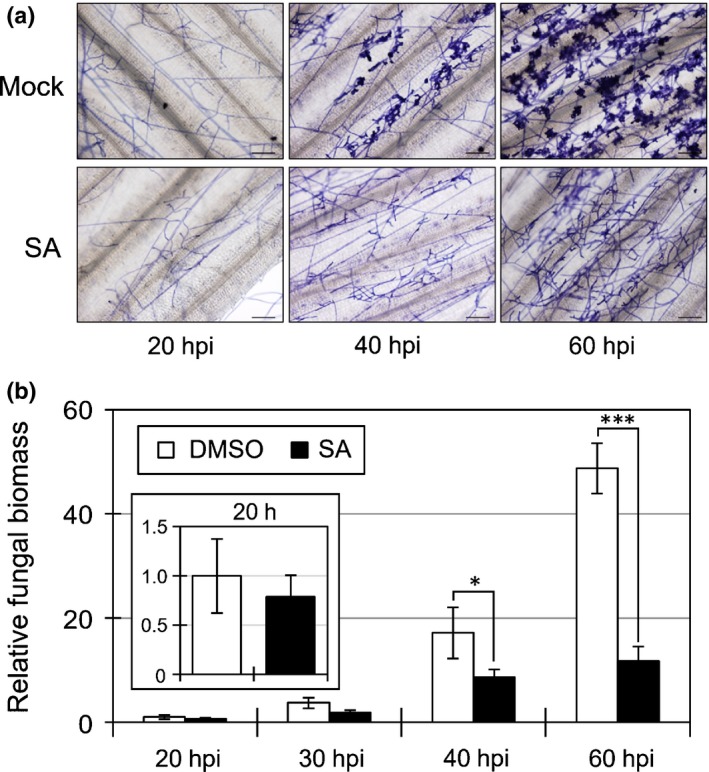
Salicylic acid (SA) induces post‐invasion resistance to *Rhizoctonia solani* in *Brachypodium distachyon*. (a) Hyphal growth of *R. solani* AG‐1 on *B. distachyon* leaf surfaces treated with dimethyl sulfoxide (DMSO; Mock) or 1 mM SA. The inoculated leaves were collected at the indicated time points and *R. solani* hyphae were stained with trypan blue. Infection cushions were recognized as aggregates of convoluted hyphae. Bars, 100 μm. (b) Relative biomass (linear scale) of *R. solani* AG‐1 in *B. distachyon* leaves treated with DMSO (Mock) or 1 mM SA at the indicated time points. Data are represented as means ± SEM,* n* = 6; *, *P* < 0.05; ***, *P* < 0.001 using Student's *t*‐tests relative to the values of mock at 20 h post‐inoculation (hpi). The experiments were repeated at least twice with similar results.

The infection behavior of *R. solani* on *B. distachyon* was monitored by both microscopic observations and fungal biomass quantification. Mycelia randomly expanded on the leaf surfaces at 20 h post‐inoculation (hpi) (Fig. 5a). At 40 hpi, the mycelia were very dense, and the specialized infection structures, termed infection cushions, began to appear (Marshall & Rush, 1980a,b; Matsuura, 1986). Many infection cushions were evident at 60 hpi. Fungal hyphae inside the inoculated leaves were detected at 20 hpi (Fig. 5b), but not at 5 hpi (Fig. S3). The number of fungal hyphae within inoculated leaves was slightly higher at 30 hpi, and then increased significantly, accompanied by the formation of infection cushions. Our findings indicate that *R. solani* invaded *B. distachyon* leaves within 20 hpi before the development of infection cushions. Previous studies have shown that *R. solani* can also penetrate the epidermis from the lobate appressorium and mycelial tip (Marshall & Rush, 1980a,b; Singh *et al*., 2003). Thus, the infection process of *R. solani* appeared to be divided into two phases. First, *R. solani* intrudes into the host by suppressing various layers of immunity using enzymes, chemicals and effectors. Next, infection cushions are formed using nutrients absorbed from the host, enabling the pathogen to penetrate large numbers of infection hyphae into the epidermal cells. After or during this second phase, toxins may be produced that shift the infection behavior of *R. solani* to the necrotrophic phase (Vidhyasekaran *et al*., 1997). Indeed, necrotic lesions and chlorosis were observed 2–3 d (48–72 h) after inoculation (Figs 1, 2a,e).

### Effect of SA‐induced resistance on *R. solani* development in *B. distachyon*

In SA‐treated plants, the mycelial density increased with time; however, interestingly, infection cushions were hardly found at the time points investigated (Fig. 5a). Further, massive increments in fungal biomass were not detected at 40 and 60 hpi; the fungal biomass was suppressed to a significantly lower level than that in the control (Fig. 5b). We repeated these experiments at least twice with the same results. Given that the fungal biomass in SA‐treated leaves was comparable with that in the control at 20 hpi (enlarged box in Fig. 5b), we speculated that SA treatment did not prevent the primary invasion of *R. solani*. Conversely, the unsuccessful formation of infection cushions suggested that the transition from the first to the second phase was inhibited by the SA‐induced resistance in *B. distachyon*.

### Effects of plant defense activators on *R. solani* resistance

**Figure 6:**
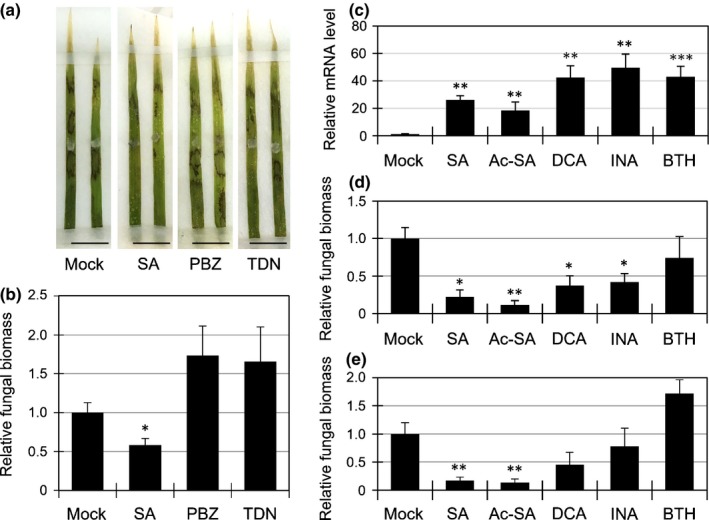
Specific salicylic acid (SA) analogs, but not commercial plant defense activators, increased *Rhizoctonia solani* resistance in *Brachypodium distachyon*. (a, b) Lesion formation (a) and relative biomass (linear scale) (b) of *R. solani* AG‐1 at 3 d post‐inoculation (dpi) in *B. distachyon* leaves treated with dimethyl sulfoxide (DMSO; Mock), 1 mM SA, 200 μM probenazole (PBZ) or 200 μM tiadinil (TDN). Bars, 1 cm. Data are represented as means ± SEM,* n *= 4; *, *P* < 0.05 using Student's *t*‐tests. (c) Expression levels of an SA‐responsive marker gene *BdWRKY45L1* (*Bradi2 g30695*). Bd21 seedlings were treated with water (Mock) or 500 μM chemical solutions for 24 h. Data are represented as means ± SEM,* n *= 3; **, *P *< 0.01; ***, *P *< 0.001 using Student's *t*‐tests. (d, e) Relative biomass (linear scale) of *R. solani* AG‐1 at 3 dpi (d) or 6 dpi (e) in *B. distachyon* leaves sprayed with DMSO (Mock) or 500 μM of SA, acetylsalicylic acid (Ac‐SA), 3,5‐dichloroanthranilic acid (DCA), 2,6‐dichlorisonicotianic acid (INA) or benzothiadiazole (BTH). Data are represented as means ± SEM,* n *= 4; *, *P *< 0.05; **, *P *< 0.01 using Student's *t*‐tests. The experiments were repeated three times with similar results.

Our finding that SA can confer disease resistance to *R. solani* is inconsistent with the fact that no commercial plant defense activators for rice blast are effective in controlling sheath blight, although all potentiate SA signaling (Schreiber & Desveaux, 2008; Noutoshi *et al*., 2012). To validate this point, we investigated the effects of probenazole and tiadinil on *R. solani* resistance in *B. distachyon*, and found that none conferred resistance (Fig. 6a,b). As these compounds used in agriculture are potentiators, but not strong inducers, of defense responses, we hypothesized that an already elevated or considerably rapid induction of SA signaling, or a particular set of SA‐specific transcripts, is required for *R. solani* resistance. To test this hypothesis, we further evaluated the effect of the following structurally or functionally related SA analogs: Ac‐SA, DCA, INA and BTH. These compounds induced the transcription of *BdWRKY45L1* (*Bradi2 g30695*), a marker gene for SA response, after 24 h of treatment in *B. distachyon* (Fig. 6c) (Kouzai *et al*., 2016). The *B. distachyon* leaves were then treated with these analogs for 24 h, followed by inoculation with *R. solani*. We found that Ac‐SA, DCA and INA suppressed fungal growth compared with that in the control treatment at 3 dpi; however, the resistance provided by DCA and INA was impaired at 6 dpi (Fig. 6d,e). Surprisingly, BTH did not induce resistance at either 3 or 6 dpi (Fig. 6d,e), although it could induce resistance in rice against blast disease and bacterial leaf blight (Shimono *et al*., 2007, 2012). These results support the idea that a previously induced defense by SA confers resistance to *R. solani*; however, the activity and durability of *R. solani* resistance varied across the compounds tested.

### Transcriptome analysis of *B. distachyon* treated with SA or BTH

**Figure 7:**
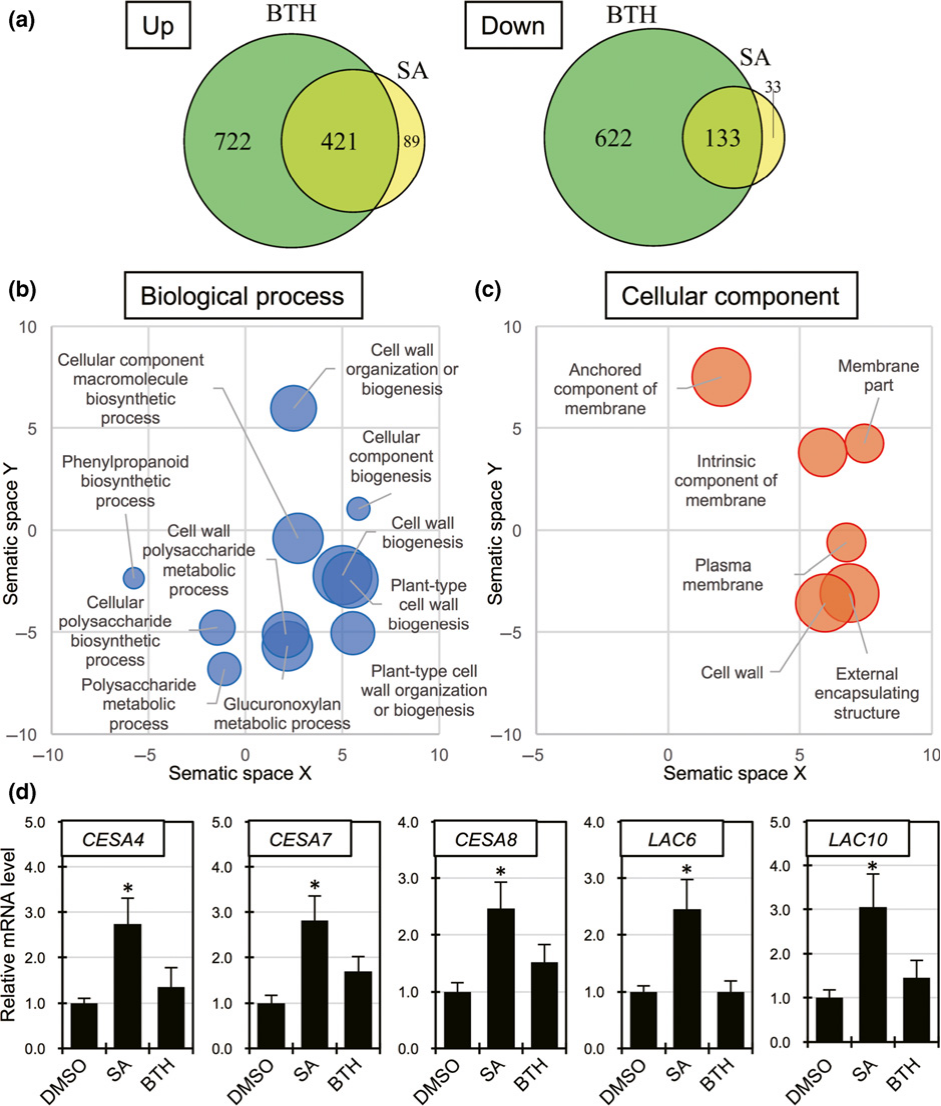
Salicylic acid (SA)‐specific transcripts include secondary cell wall‐related genes in *Brachypodium distachyon*. (a) Proportional Venn diagrams showing the overlap of the up‐regulated (left) and down‐regulated (right) gene sets 24 h after treatment with SA or benzothiadiazole (BTH). (b, c) Functional classification of the genes specifically upregulated by SA with gene ontology (GO) categories related to biological process (b) and cellular component (c) using a REVIGO scatterplot. Circles denote significantly enriched GO terms with false discovery rate (FDR) < 0.05. The circle size represents the −log_10_ transformed FDR in REVIGO analysis. (d) Expression levels of secondary cell wall‐related genes after 24 h of treatment with SA or BTH. Data are represented as means ± SEM relative to those of the dimethyl sulfoxide (DMSO) treatment, *n *= 3; *, *P *< 0.05 using Student's *t*‐tests. All experiments were repeated at least twice with similar results.

To understand the reason why SA, but not BTH, induced resistance in *B. distachyon* to *R. solani*, we compared the datasets of the transcriptome of Bd21 leaves after SA treatment with those after BTH treatment, and identified the genes that were differentially expressed in each treatment. Through our RNA‐seq analysis of three biological replicates with SA, BTH and Mock (DMSO), 94.7% of the sequencing reads were mapped to the reference genome of *B. distachyon* Bd21 on average (Table S2). Then, we identified 676 and 1898 genes as DEGs in response to SA and BTH, respectively (Tables S3, S4). The degrees of overlap of these DEGs in both up‐ and down‐regulated genes are depicted in Fig. 7(a); 82.0% of the DEGs in the SA treatment were shared with those in the BTH treatment, indicating that BTH acts as a functional SA analog in *B. distachyon*, as in Arabidopsis and rice (Fig. 7a). However, we found that 89 genes were induced by SA only (Fig. 7a). The GO enrichment analysis demonstrated that GO terms related to the biosynthesis of the secondary cell wall (SCW) were clearly over‐represented in the given gene set (Fig. 7b,c). We found that at least 17 genes (20.7%) showed homology to Arabidopsis genes with a GO term (GO:0042546) related to cell wall biogenesis (Table S5). In *B. distachyon, BdCESA4*,* 7* and *8* encode cellulose synthase A (CESA) proteins and the loss‐of‐function mutants for *BdCESA4* and *7* showed a reduced amount of crystalline cellulose in the SCW (Handakumbura *et al*., 2013). *BdLAC5*,* 6* and *10* are members of the laccase family and mutation in *BdLAC5* leads to a reduction in the culm lignin level (Wang *et al*., 2015). In our RNA‐seq analysis, *BdLAC5* was induced by both SA and BTH, but *BdLAC6* and *10* were found in the SA‐specific DEGs. The expression profiles of *BdCESA4*,* 7* and *8*, and *BdLAC6* and *10*, were evaluated by qRT‐PCR analysis, and were shown to be induced by SA, but not BTH, thus confirming the transcriptome data (Fig. 7d). These results imply that SCW synthesis and reinforcement are induced by SA, which may act as a critical cellular response in SA‐induced resistance against *R. solani* infection in *B. distachyon*. The number of DEGs for BTH is 2.8 times greater than that for SA, and > 1300 genes were specifically regulated by BTH at an equal concentration to the SA treatment (Fig. 7a); therefore, we cannot exclude the possibility that these BTH‐specific genes compromise the defense responses to *R. solani*.

### Sheath blight resistance accession of *B. distachyon* against *R. solani*

**Figure 8:**
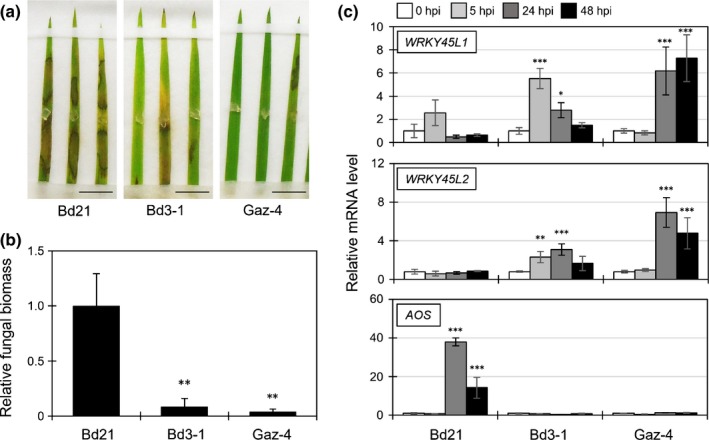
*Brachypodium distachyon* accessions Bd3‐1 and Gaz‐4 are resistant to *Rhizoctonia solani*. (a, b) Lesion formation (a) and relative biomass (linear scale) (b) of *R. solani* AG‐1 in the leaves of *B. distachyon* accessions Bd21, Bd3‐1 and Gaz‐4 at 3 d post‐inoculation (dpi). Bars, 1 cm. Data are represented as means ± SEM,* n* = 5; **, *P* < 0.01 using Student's *t*‐test. (c) Expression levels of *B. distachyon* marker genes *BdWRKY45L1* and *BdWRKY45L2* for SA and *BdAOS* for jasmonic acid (JA) in Bd21, Bd3‐1 and Gaz‐4 at 0, 5, 24 and 48 h post‐inoculation (hpi) with *R. solani* AG‐1. Data are represented as means ± SEM,* n *= 4; *, *P *< 0.05; **, *P *< 0.01; *P *< 0.001 using Student's *t‐*tests compared with the corresponding values of each accession at 0 dpi. All experiments were repeated at least three times with similar results.

For host resistance often found in a cultivar‐specific trait, plants recognize pathogen‐derived effector proteins using resistance (R) proteins and activate robust immune responses termed effector‐triggered immunity (ETI) (Jones & Dangl, 2006), in which SA plays an essential role (Vlot *et al*., 2009). From the *B. distachyon* germplasm collections, we searched for *R. solani*‐resistant accessions and identified Bd3‐1 and Gaz‐4 (Vogel *et al*., 2006, 2009). Our results confirmed the recent finding that the Bd3‐1 accession is resistant to *R. solani* RhBC 2‐1‐35; however, the AG of this isolate has not been reported (Sandoya & Buanafina, 2014). The lesions and accumulated fungal biomasses in leaves of Bd3‐1 and Gaz‐4, caused by infection with *R. solani* AG‐1, were significantly less than those in Bd21 (Fig. 8a,b). During the infection of *R. solani*, the SA marker genes *BdWRKY45L1* and *BdWRKY45L2* were transcribed within 5 and 24 hpi in Bd3‐1 and Gaz‐4, respectively, but not in Bd21 (Fig. 8c) (Kouzai *et al*., 2016), suggesting the activation of ETI in these *R. solani*‐resistant accessions. By contrast, the JA marker *BdAOS* was clearly induced only in Bd21 (Kouzai *et al*., 2016). The quantitative measurement of endogenous phytohormones during infection showed that SA levels were not altered by fungal inoculation in either accession, but JA and JA‐Ile significantly accumulated only in Bd21 (Fig. S4). The levels of other phytohormones, such as auxin, abscisic acid and cytokinins, did not change in any accession (Fig. S4). The elevated JA levels and JA marker gene expression were well correlated in the susceptible Bd21. These findings could be a result of the suppression of SA signaling by the pathogen and the necrotic lesions recognized as wounding.

## Discussion

In this study, we found that foliar pretreatments with SA in *B. distachyon* and rice prevented symptom development caused by *R. solani* (Figs 2a,b, 4a,b). The fungal biomass of *R. solani* in the infected leaves was maintained at a lower level by SA pretreatment compared with that of non‐treated leaves, indicating that SA can induce resistance, at least in these plant species, against *R. solani* (Fig. 5). However, *NahG* transgenic rice plants which showed decreased SA levels became more susceptible to *R. solani* (Fig. 4c,d). These results suggest that *R. solani* probably uses the biotrophic stage in a short window of time for early infection; SA‐induced defense responses could attenuate the infection at this point to combat the disease. This result is consistent with the recent finding that *R. solani* expresses secreted proteinaceous effectors during infection as potential virulence factors (Zheng *et al*., 2013; Anderson *et al*., 2016). The JA‐induced susceptibility of *B. distachyon* to *R. solani* might be attributed to the antagonistic interaction between SA and JA, based on observations mainly in Arabidopsis (Kunkel & Brooks, 2002). However, JA did not decrease resistance to *P. oryzae* (Fig. S2). As SA and JA share a certain range of defense‐related genes, designated as the common defense system in rice, unlike in dicots (Tamaoki *et al*., 2013 [Correction added after online publication 19 October 2017: the citation Tamaoki *et al*. (2013) has been corrected here and in the References list]; De Vleesschauwer *et al*., 2014; Kouzai *et al*., 2016), the results might depend on the degree of contribution of this common system to resistance against *R. solani* and *P. oryzae*.

*Rhizoctonia solani* might possess complicated virulence mechanisms that include not only chemical or enzymatic toxins, but also effector proteins, to suppress host immunity. *Rhizoctonia solani* has been isolated from many infested field crops showing various necrotic symptoms, but the host range of each isolate seems to be limited and is dependent on the virulence related to the AGs (Anderson, 1982; Ogoshi, 1987). For example, *A. thaliana* is susceptible to *R. solani* AG‐2‐1 and AG‐4 HG‐I, but not to AG‐8 (Sharon *et al*., 2011; Foley *et al*., 2013), and the different types of AGs of *R. solani* exhibit varying infectivity to cauliflower (Pannecoucque & Hofte, 2009). The host specificity of *R. solani*, considering the effector proteins in addition to toxins, should provide new insights into the infection strategy of this pathogen, although the sensitivity to pathogen‐produced toxins also contributes strongly to symptom development in rice (Brooks, 2007).

The formation of infection cushions of *R. solani* was clearly suppressed by SA pretreatment in *B. distachyon* (Fig. 5). The relationships between infection cushions or lobate appressoria and disease severity have been investigated using various rice cultivars with varying susceptibility levels (Marshall & Rush, 1980b). The resistance level and infection structure numbers were correlated and, interestingly, infection cushions, but not lobate appressoria, were not observed in resistant rice cultivars. Further, a relationship is known to exist between the endogenous SA level and blast resistance in rice cultivars (Silverman *et al*., 1995). From the data demonstrated in these two previous reports, we noted that the *R. solani*‐resistant cultivar Tetep containing high levels of SA was resistant to rice blast, whereas the *R. solani* very susceptible cultivar Labelle containing low SA levels was susceptible (Marshall & Rush, 1980b; Silverman *et al*., 1995). The endogenous SA content could also be related to the sheath blight susceptibility in rice cultivars.

*Rhizoctonia solani*‐resistant rice cultivars, but not susceptible ones, deposit cuticular wax on the outer sheath surface, and the removal of wax in resistant cultivars reduced the resistance level (Marshall & Rush, 1980a). Wax production may be regulated by SA signaling. It has also been demonstrated that the exogenous application of glucose or 3‐o‐methylglucose (MEG) confers disease resistance in rice and cotton against *R. solani* (Weinhold & Bowman, 1974; Marshall & Rush, 1980a). As some types of rare sugar have also been found to induce disease resistance to blast and bacterial blight in rice, such sugar‐induced defense responses might also be effective against sheath blight disease (Kano *et al*., 2010, 2011).

We have shown that SA, Ac‐SA and INA, but not probenazole, tiadinil and BTH, confer resistance to *R. solani* in *B. distachyon* (Fig. 6). This variation might depend on certain chemical properties, such as the affinity to receptors and rates of incorporation and metabolism in plants. For instance, the DCA‐induced transcriptional profile is different from that of SA in Arabidopsis (Knoth *et al*., 2009). SA is known to induce thermogenesis in voodoo lily, and only two of the 33 SA analogs can induce this response (Vlot *et al*., 2009). The immunity contributing to *R. solani* resistance might be attributed to a specific set of defense responses or the appropriate induction timing of SA signaling. The difference in these synthetic compounds from SA provides an opportunity to identify which type of plant defense response can function for the inhibition of *R. solani* infection. Transcriptome analysis after treatment with SA and BTH in *B. distachyon* leaves revealed a difference between these two compounds (Fig. 7a). In Arabidopsis, 89% and 99% of the genes transcriptionally induced by SA and BTH, respectively, were dependent on *NPR1*, indicating the high overlap between them (Wang *et al*., 2006; Blanco *et al*., 2009). However, in *B. distachyon*, BTH regulated 82% of the SA‐related DEGs, but corresponded to only 29.2% of BTH‐related DEGs. BTH could affect various developmental and physiological phenomena in *B. distachyon* compared with those in *A. thaliana*. 18.0% of SA‐related DEGs were independent of BTH and 89 genes (17.5% of SA‐induced genes) were specifically upregulated by SA. The GO enrichment analysis showed the over‐representation of SCW‐related GO terms in this gene set (Fig. 7b,c; Table S5). SCW is known to play a central role as a physical barrier against pathogens, and SCW reinforcement, such as lignification, is induced in response to pathogen infection as a defense response (Miedes *et al*., 2014). Recently, the resistance gene *Xa4*, which encodes a cell wall‐associated kinase, has been reported to confer race‐specific resistance to a bacterial pathogen *Xanthomonas oryzae* pv. *oryzae* (*Xoo*) via the promotion of SCW reinforcement in rice, and the *cesa4* rice mutant, as well as the RNAi transgenic rice lines for *OsCESA4*,* 7* and *9*, compromised the *Xa4*‐mediated race‐specific resistance to *Xoo* (Hu *et al*., 2017). From the culture filtrates of vegetative and infection samples of *R. solani* AG‐8, many proteins with predicted function relating to lignin catabolism were detected, and were thought to be involved in the modification of the host cell wall (Anderson *et al*., 2016). Therefore, SCW reinforcement might be a crucial event in SA‐induced *R. solani* resistance in *B. distachyon*. Further studies are needed to clarify the contribution of wall strengthening to *R. solani* resistance.

In rice, six quantitative trait loci (QTLs) contributing to resistance to *R. solani* were identified using siblings between susceptible Lemont and resistant Tequing cultivars (Li *et al*., 1995). The mapped chromosomal regions were associated with QTLs responsible for plant height and subsequent heading. Another group identified QTLs for sheath blight resistance in the rice line derived from the resistant Tetep variety; these QTLs were also associated with culm length and heading date (Sato *et al*., 2004). These results suggest that *R. solani* resistance in rice strongly relies on morphological or developmental phenotypes, especially during field evaluation. By contrast, the sheath blight resistance of *B. distachyon* accessions Bd3‐1 and Gaz‐4, found in this study, seemed to be associated with true resistance, as it clearly activated SA‐dependent signaling after inoculation of *R. solani* (Fig. 8c). As activation was detected within 24 h, these accessions seem to recognize infection of *R. solani* at an early time point after inoculation. Interestingly, Bd3‐1 was also resistant to *R. solani* AG‐5 (data not shown), suggesting that Bd3‐1 recognizes the common factor between the different AGs of *R. solani* isolates or possesses another resistance gene for AG‐5. Further studies to identify plant genes that confer resistance or tolerance to *R. solani* might elucidate the mechanisms of how plants develop resistance to *R. solani*, as well as the molecular infection strategy of this pathogen.

In the resistant Bd3‐1 accession, the SA marker genes were highly expressed, but SA levels were not upregulated (Fig. S4). This finding is similar to the situation reported in rice, in which the SA levels were not changed locally and systemically even after challenge with an incompatible pathogen, but an SA marker gene *OsWRKY45* was upregulated (Jiang *et al*., 2010; Takatsuji, 2014). Furthermore, SA accumulation did not occur in barley in response to an incompatible powdery mildew fungus, although an HR cell death was observed at the infection sites (Huckelhoven *et al*., 1999). Unlike in dicotyledonous plants (Vlot *et al*., 2009; Noutoshi *et al*., 2012), activation of SA signaling might not be associated with the obvious accumulation of free SA in monocots.

The present study provides the means to produce crop protection against sheath blight disease with environmental sustainability and economy. The first strategy is to identify disease resistance inducers. Plant defense‐activating compounds with SA‐related functionality could be developed as an alternative method for *R. solani*. As our results suggest the importance of the activation of SCW biosynthesis for the resistance to *R. solani* (Fig. 7b,c; Table S5), the activity for the transcriptional induction of the 89 genes including SCW‐related genes may be required for the potential candidate chemicals. The second strategy is the development of disease‐resistant/tolerant cultivars. Recently, disease resistance genes have been explored from wild relatives or closely related species to complement the depletion or limitation of genetic resources for disease resistance in cultivated crops (Jones *et al*., 2014; Du *et al*., 2015; Kawashima *et al*., 2016). *Rhizoctonia solani* is also a causal agent of root rot diseases in various plant species, and wheat cultivars and *B. distachyon* accessions partially resistant to *R. solani* AG‐8 have been identified (Mahoney *et al*., 2016; Schneebeli *et al*., 2016). The disease‐resistant genes identified in *B. distachyon* can be used for the molecular breeding of sheath blight resistance in rice and probably to increase resistance to various *Rhizoctonia* diseases in other crops.

## Author contributions

Y.K., H.M., M.Y., Y.I., K.T., E.M, Y. Nishizawa and Y. Noutoshi conceived the study and designed the experiments. Y.K., M.K., M.W., K.K. and Y. Noutoshi carried out the experiments and performed the statistical analysis, except as noted below. Y.K., K.T. and D.O. performed the experiment shown in Fig. 2(e,f). T.M., I.C.M. and T.H. carried out phytohormone measurements. K.I. and K.M. carried out the RNA‐seq analysis and data deposition on the DDBJ Sequence Read Archive. Y.K., M.K., E.M. and Y. Nishizawa performed the inoculation tests using rice. Y.K. and T.S. performed the microscopic analysis. Y.K. and Y. Noutoshi drafted the manuscript. H.M., M.Y., Y.I., K.T., Y.O. and K.M. contributed to the analysis, interpretation of the data and critical revision of the manuscript.

## Supporting information

Please note: Wiley Blackwell are not responsible for the content or functionality of any Supporting Information supplied by the authors. Any queries (other than missing material) should be directed to the *New Phytologist* Central Office.

**Fig. S1** Hyphal growth of *Rhizoctonia solani* on nutrient agar medium containing phytohormones.
**Fig. S2** Effects of phytohormones on *Brachypodium distachyon* disease resistance to *Pyricularia oryzae* and *Botrytis cinerea*.
**Fig. S3** *Rhizoctonia solani* biomass in *Brachypodium distachyon* leaves at the initial infection stage.
**Fig. S4** Endogenous levels of phytohormones in *Brachypodium distachyon* accessions Bd21 and Bd3‐1 after inoculation with *Rhizoctonia solani*.
**Table S1** Primers used in this study
**Table S2** Summary of the sequence reads from RNA‐seq analysis mapped to the *Brachypodium distachyon* Bd21 genome
**Table S3** Differentially expressed genes (DEGs) of *Brachypodium distachyon* in salicylic acid treatment
**Table S4** Differentially expressed genes (DEGs) of *Brachypodium distachyon* in benzothiadiazole treatment
**Table S5** Cell wall biogenesis (GO:0042546)‐related genes in the differentially expressed genes (DEGs) specifically induced by salicylic acid

## References

[nph14849-bib-0001] Anderson J , Hane J , Stoll T , Pain N , Hastie M , Kaur P , Hoogland C , Gorman J , Singh K . 2016 Proteomic analysis of *Rhizoctonia solani* identifies infection‐specific, redox associated proteins and insight into adaptation to different plant hosts. Molecular & Cellular Proteomics 15: 1188–1203. 26811357 10.1074/mcp.M115.054502 PMC4824849

[nph14849-bib-0002] Anderson N . 1982 The genetics and pathology of *Rhizoctonia solani* . Annual Review of Phytopathology 20: 329–347.

[nph14849-bib-0003] Blanco F , Salinas P , Cecchini N , Jordana X , Van Hummelen P , Alvarez M , Holuigue L . 2009 Early genomic responses to salicylic acid in Arabidopsis. Plant Molecular Biology 70: 79–102. 19199050 10.1007/s11103-009-9458-1

[nph14849-bib-0004] Brooks S . 2007 Sensitivity to a phytotoxin from *Rhizoctonia solani* correlates with sheath blight susceptibility in rice. Phytopathology 97: 1207–1212. 18943678 10.1094/PHYTO-97-10-1207

[nph14849-bib-0005] Budge G , Shaw M , Colyer A , Pietravalle S , Boonham N . 2009 Molecular tools to investigate *Rhizoctonia solani* distribution in soil. Plant Pathology 58: 1071–1080.

[nph14849-bib-0006] De Vleesschauwer D , Xu J , Hofte M . 2014 Making sense of hormone‐mediated defense networking: from rice to Arabidopsis. Frontiers in Plant Science 5: 611. 25426127 10.3389/fpls.2014.00611 PMC4227482

[nph14849-bib-0007] Du J , Verzaux E , Chaparro‐Garcia A , Bijsterbosch G , Keizer LCP , Zhou J , Liebrand TWH , Xie C , Govers F , Robatzek S *et al* 2015 Elicitin recognition confers enhanced resistance to *Phytophthora infestans* in potato. Nature Plants 1: 15034. 27247034 10.1038/nplants.2015.34

[nph14849-bib-0008] Ebbole D . 2007 Magnaporthe as a model for understanding host–pathogen interactions. Annual Review of Phytopathology 45: 437–456. 10.1146/annurev.phyto.45.062806.094346 17489691

[nph14849-bib-0009] Ferrari S , Plotnikova J , De Lorenzo G , Ausubel F . 2003 *Arabidopsis* local resistance to *Botrytis cinerea* involves salicylic acid and camalexin and requires *EDS4* and *PAD2*, but not *SID2*,* EDS5* or *PAD4* . Plant Journal 35: 193–205. 12848825 10.1046/j.1365-313x.2003.01794.x

[nph14849-bib-0010] Fitzgerald T , Powell J , Schneebeli K , Hsia M , Gardiner D , Bragg J , McIntyre C , Manners J , Ayliffe M , Watt M *et al* 2015 *Brachypodium* as an emerging model for cereal–pathogen interactions. Annals of Botany 115: 717–731. 25808446 10.1093/aob/mcv010 PMC4373291

[nph14849-bib-0011] Foley R , Gleason C , Anderson J , Hamann T , Singh K . 2013 Genetic and genomic analysis of *Rhizoctonia solani* interactions with *Arabidopsis*; evidence of resistance mediated through NADPH oxidases. PLoS ONE 8: e56814. 23451091 10.1371/journal.pone.0056814 PMC3581538

[nph14849-bib-0012] Glazebrook J . 2005 Contrasting mechanisms of defense against biotrophic and necrotrophic pathogens. Annual Review of Phytopathology 43: 205–227. 10.1146/annurev.phyto.43.040204.135923 16078883

[nph14849-bib-0013] Govrin E , Levine A . 2002 Infection of Arabidopsis with a necrotrophic pathogen, *Botrytis cinerea*, elicits various defense responses but does not induce systemic acquired resistance (SAR). Plant Molecular Biology 48: 267–276. 11855728 10.1023/a:1013323222095

[nph14849-bib-0014] Handakumbura P , Matos D , Osmont K , Harrington M , Heo K , Kafle K , Kim S , Baskin T , Hazen S . 2013 Perturbation of *Brachypodium distachyon CELLULOSE SYNTHASE A4* or *7* results in abnormal cell walls. BMC Plant Biology 13: 131. 24024469 10.1186/1471-2229-13-131 PMC3847494

[nph14849-bib-0015] Hashiba T . 1984 Estimating method of severity and yield loss by rice sheath blight disease. Bulletin of the Hokuriku National Agricultural Experiment Station 26: 115–164.

[nph14849-bib-0016] Helliwell E , Wang Q , Yang Y . 2013 Transgenic rice with inducible ethylene production exhibits broad‐spectrum disease resistance to the fungal pathogens *Magnaporthe oryzae* and *Rhizoctonia solani* . Plant Biotechnology Journal 11: 33–42. 23031077 10.1111/pbi.12004

[nph14849-bib-0017] Hu K , Cao J , Zhang J , Xia F , Ke Y , Zhang H , Xie W , Liu H , Cui Y , Cao Y *et al* 2017 Improvement of multiple agronomic traits by a disease resistance gene via cell wall reinforcement. Nature Plants 3: 17009. 28211849 10.1038/nplants.2017.9

[nph14849-bib-0018] Huckelhoven R , Fodor J , Preis C , Kogel K . 1999 Hypersensitive cell death and papilla formation in barley attacked by the powdery mildew fungus are associated with hydrogen peroxide but not with salicylic acid accumulation. Plant Physiology 119: 1251–1260. 10198083 10.1104/pp.119.4.1251 PMC32009

[nph14849-bib-0019] Jiang C , Shimono M , Sugano S , Kojima M , Yazawa K , Yoshida R , Inoue H , Hayashi N , Sakakibara H , Takatsuji H . 2010 Abscisic acid interacts antagonistically with salicylic acid signaling pathway in rice–*Magnaporthe grisea* interaction. Molecular Plant–Microbe Interactions 23: 791–798. 20459318 10.1094/MPMI-23-6-0791

[nph14849-bib-0020] Jones J , Dangl J . 2006 The plant immune system. Nature 444: 323–329. 17108957 10.1038/nature05286

[nph14849-bib-0021] Jones J , Witek K , Verweij W , Jupe F , Cooke D , Dorling S , Tomlinson L , Smoker M , Perkins S , Foster S . 2014 Elevating crop disease resistance with cloned genes. Philosophical Transactions of the Royal Society B: Biological Sciences 369: 20130087. 10.1098/rstb.2013.0087 PMC3928893 24535396

[nph14849-bib-0022] Kano A , Gomi K , Yamasaki‐Kokudo Y , Satoh M , Fukumoto T , Ohtani K , Tajima S , Izumori K , Tanaka K , Ishida Y *et al* 2010 A rare sugar, D‐Allose, confers resistance to rice bacterial blight with upregulation of defense‐related genes in *Oryza sativa* . Phytopathology 100: 85–90. 19968553 10.1094/PHYTO-100-1-0085

[nph14849-bib-0023] Kano A , Hosotani K , Gomi K , Yamasaki‐Kokudo Y , Shirakawa C , Fukumoto T , Ohtani K , Tajima S , Izumori K , Tanaka K *et al* 2011 D‐Psicose induces upregulation of defense‐related genes and resistance in rice against bacterial blight. Journal of Plant Physiology 168: 1852–1857. 21601944 10.1016/j.jplph.2011.04.003

[nph14849-bib-0024] Kawashima C , Guimaraes G , Nogueira S , MacLean D , Cook D , Steuernagel B , Baek J , Bouyioukos C , Melo B , Tristao G *et al* 2016 A pigeonpea gene confers resistance to Asian soybean rust in soybean. Nature Biotechnology 34: 661–665. 10.1038/nbt.3554 27111723

[nph14849-bib-0025] Kellogg E . 2015 *Brachypodium distachyon* as a genetic model system. Annual Review of Genetics 49: 1–20. 10.1146/annurev-genet-112414-055135 26393966

[nph14849-bib-0026] Kim D , Pertea G , Trapnell C , Pimentel H , Kelley R , Salzberg S . 2013 TopHat2: accurate alignment of transcriptomes in the presence of insertions, deletions and gene fusions. Genome Biology 14: R36. 23618408 10.1186/gb-2013-14-4-r36 PMC4053844

[nph14849-bib-0027] Knoth C , Salus M , Girke T , Eulgem T . 2009 The synthetic elicitor 3,5‐dichloroanthranilic acid induces NPR1‐dependent and NPR1‐independent mechanisms of disease resistance in Arabidopsis. Plant Physiology 150: 333–347. 19304930 10.1104/pp.108.133678 PMC2675713

[nph14849-bib-0028] Kouzai Y , Kimura M , Yamanaka Y , Watanabe M , Matsui H , Yamamoto M , Ichinose Y , Toyoda K , Onda Y , Mochida K *et al* 2016 Expression profiling of marker genes responsive to the defence‐associated phytohormones salicylic acid, jasmonic acid and ethylene in *Brachypodium distachyon* . BMC Plant Biology 16: 59. 26935959 10.1186/s12870-016-0749-9 PMC4776424

[nph14849-bib-0029] Kunkel B , Brooks D . 2002 Cross talk between signaling pathways in pathogen defense. Current Opinion in Plant Biology 5: 325–331. 12179966 10.1016/s1369-5266(02)00275-3

[nph14849-bib-0030] Langmead B , Salzberg S . 2012 Fast gapped‐read alignment with Bowtie 2. Nature Methods 9: 357–359. 22388286 10.1038/nmeth.1923 PMC3322381

[nph14849-bib-0031] Lee F , Rush M . 1983 Rice sheath blight: a major rice disease. Plant Disease 67: 829–832.

[nph14849-bib-0032] Li Z , Pinson S , Marchetti M , Stansel J , Park W . 1995 Characterization of quantitative trait loci (QTLs) in cultivated rice contributing to field resistance to sheath blight (*Rhizoctonia solani*). Theoretical and Applied Genetics 91: 382–388. 24169789 10.1007/BF00220903

[nph14849-bib-0033] Mahoney A , Babiker E , Paulitz T , See D , Okubara P , Hulbert S . 2016 Characterizing and mapping resistance in synthetic‐derived wheat to *Rhizoctonia* root rot in a green bridge environment. Phytopathology 106: 1170–1176. 27349737 10.1094/PHYTO-02-16-0055-FI

[nph14849-bib-0034] Marshall D , Rush M . 1980a Infection cushion formation on rice sheaths by *Rhizoctonia solani* . Phytopathology 70: 947–950.

[nph14849-bib-0035] Marshall D , Rush M . 1980b Relation between infection by *Rhizoctonia solani* and *R. oryzae* and disease severity in rice. Phytopathology 70: 941–946.

[nph14849-bib-0036] Matsuura K . 1986 Scanning electron microscopy of the infection process of *Rhizoctonia solani* in leaf sheaths of rice plants. Phytopathology 76: 811–814.

[nph14849-bib-0037] McCarthy D , Chen Y , Smyth G . 2012 Differential expression analysis of multifactor RNA‐Seq experiments with respect to biological variation. Nucleic Acids Research 40: 4288–4297. 22287627 10.1093/nar/gks042 PMC3378882

[nph14849-bib-0038] Miedes E , Vanholme R , Boerjan W , Molina A . 2014 The role of the secondary cell wall in plant resistance to pathogens. Frontiers in Plant Science 5: 358. 25161657 10.3389/fpls.2014.00358 PMC4122179

[nph14849-bib-0039] Mikami K , Mori IC , Matsuura T , Ikeda Y , Kojima M , Sakakibara H , Hirayama T . 2016 Comprehensive quantification and genome survey reveal the presence of novel phytohormone action modes in red seaweeds. Journal of Applied Phycology 28: 2539–2548.

[nph14849-bib-0040] Mochida K , Uehara‐Yamaguchi Y , Takahashi F , Yoshida T , Sakurai T , Shinozaki K . 2013 Large‐scale collection and analysis of full‐length cDNAs from *Brachypodium distachyon* and integration with Pooideae sequence resources. PLoS ONE 8: e75265. 24130698 10.1371/journal.pone.0075265 PMC3793998

[nph14849-bib-0041] Noutoshi Y , Okazaki M , Kida T , Nishina Y , Morishita Y , Ogawa T , Suzuki H , Shibata D , Jikumaru Y , Hanada A *et al* 2012 Novel plant immune‐priming compounds identified via high‐throughput chemical screening target salicylic acid glucosyltransferases in Arabidopsis. Plant Cell 24: 3795–3804. 22960909 10.1105/tpc.112.098343 PMC3480303

[nph14849-bib-0042] Ogoshi A . 1987 Ecology and pathogenicity of anastomosis and intraspecific groups of *Rhizoctonia solani* Kühn. Annual Review of Phytopathology 25: 125–143.

[nph14849-bib-0043] Olaya G , Buitrago C , Pearsaul D , Sierotzki H , Tally A . 2012 Detection of resistance to QoI fungicides in *Rhizoctonia solani* isolates from rice. Phytopathology 102: S4.88(Abstr).

[nph14849-bib-0044] Pannecoucque J , Hofte M . 2009 Interactions between cauliflower and *Rhizoctonia* anastomosis groups with different levels of aggressiveness. BMC Plant Biology 9: 95. 19622152 10.1186/1471-2229-9-95 PMC2719643

[nph14849-bib-0045] Peng X , Hu Y , Tang X , Zhou P , Deng X , Wang H , Guo Z . 2012 Constitutive expression of rice *WRKY30* gene increases the endogenous jasmonic acid accumulation, *PR* gene expression and resistance to fungal pathogens in rice. Planta 236: 1485–1498. 22798060 10.1007/s00425-012-1698-7

[nph14849-bib-0046] Sandoya G , Buanafina M . 2014 Differential responses of *Brachypodium distachyon* genotypes to insect and fungal pathogens. Physiological and Molecular Plant Pathology 85: 53–64.

[nph14849-bib-0047] Sato H , Ideta O , Ando I , Kunihiro Y , Hirabayashi H , Iwano M , Miyasaka A , Nemoto H , Imbe T . 2004 Mapping QTLs for sheath blight resistance in the rice line WSS2. Breeding Science 54: 265–271.

[nph14849-bib-0048] Sayler R , Yang Y . 2007 Detection and quantification of *Rhizoctonia solani* AG‐1 IA, the rice sheath blight pathogen, in rice using real‐time PCR. Plant Disease 91: 1663–1668. 10.1094/PDIS-91-12-1663 30780615

[nph14849-bib-0049] Schneebeli K , Mathesius U , Zwart A , Bragg J , Vogel J , Watt M . 2016 *Brachypodium distachyo*n genotypes vary in resistance to *Rhizoctonia solani* AG8. Functional Plant Biology 43: 189–198. 10.1071/FP15244 32480452

[nph14849-bib-0050] Schreiber K , Desveaux D . 2008 Message in a bottle: chemical biology of induced disease resistance in plants. Plant Pathology Journal 24: 245–268.

[nph14849-bib-0051] Sharon M , Freeman S , Sneh B . 2011 Assessment of resistance pathways induced in *Arabidopsis thaliana* by hypovirulent *Rhizoctonia* spp. isolates. Phytopathology 101: 828–838. 21385012 10.1094/PHYTO-09-10-0247

[nph14849-bib-0052] Shimono M , Koga H , Akagi A , Hayashi N , Goto S , Sawada M , Kurihara T , Matsushita A , Sugano S , Jiang C *et al* 2012 Rice WRKY45 plays important roles in fungal and bacterial disease resistance. Molecular Plant Pathology 13: 83–94. 21726399 10.1111/j.1364-3703.2011.00732.x PMC6638719

[nph14849-bib-0053] Shimono M , Sugano S , Nakayama A , Jiang C , Ono K , Toki S , Takatsuji H . 2007 Rice WRKY45 plays a crucial role in benzothiadiazole‐inducible blast resistance. Plant Cell 19: 2064–2076. 17601827 10.1105/tpc.106.046250 PMC1955718

[nph14849-bib-0054] Silverman P , Sseskar M , Kanter D , Schweizer P , Metraux J , Raskin I . 1995 Salicylic acid in rice – biosynthesis, conjugation, and possible role. Plant Physiology 108: 633–639. 12228500 10.1104/pp.108.2.633 PMC157383

[nph14849-bib-0055] Singh A , Rohiln R , Savary S , Willocquet L , Singh US . 2003 Infection process in sheath blight of rice caused by *Rhizoctonia solan* . Indian Phytopathology 56: 434–438.

[nph14849-bib-0056] Soreng R , Peterson P , Romaschenko K , Davidse G , Zuloaga F , Judziewicz E , Filgueiras T , Davis J , Morrone O . 2015 A worldwide phylogenetic classification of the Poaceae (Gramineae). Journal of Systematics and Evolution 53: 117–137.

[nph14849-bib-0057] Spoel SH , Johnson JS , Dong X . 2007 Regulation of tradeoffs between plant defenses against pathogens with different lifestyles. Proceedings of the National Academy of Sciences, USA 104: 18842–18847. 10.1073/pnas.0708139104 PMC2141864 17998535

[nph14849-bib-0058] Takatsuji H . 2014 Development of disease‐resistant rice using regulatory components of induced disease resistance. Frontiers in Plant Science 5: 630. 25431577 10.3389/fpls.2014.00630 PMC4230042

[nph14849-bib-0059] Tamaoki D , Seo S , Yamada S , Kano A , Miyamoto A , Shishido H , Miyoshi S , Taniguchi S , Akimitsu K , Gomi K . 2013 Jasmonic acid and salicylic acid activate a common defense system in rice. Plant Signaling & Behavior 8: e24260. 23518581 10.4161/psb.24260 PMC3906320

[nph14849-bib-0060] Thomma BP , Eggermont K , Tierens KF , Broekaert WF . 1999 Requirement of functional ethylene‐insensitive 2 gene for efficient resistance of Arabidopsis to infection by *Botrytis cinerea* . Plant Physiology 121: 1093–1102. 10594097 10.1104/pp.121.4.1093 PMC59477

[nph14849-bib-0061] Vidhyasekaran P , Ponmalar T , Samiyappan R , Velazhahan R , Vimala R , Ramanathan A , Paranidharan V , Muthukrishnan S . 1997 Host‐specific toxin production by *Rhizoctonia solani*, the rice sheath blight pathogen. Phytopathology 87: 1258–1263. 18945027 10.1094/PHYTO.1997.87.12.1258

[nph14849-bib-0062] Vlot A , Dempsey D , Klessig D . 2009 Salicylic acid, a multifaceted hormone to combat disease. Annual Review of Phytopathology 47: 177–206. 10.1146/annurev.phyto.050908.135202 19400653

[nph14849-bib-0063] Vogel J , Garvin D , Leong O , Hayden D . 2006 Agrobacterium‐mediated transformation and inbred line development in the model grass *Brachypodium distachyon* . Plant Cell Tissue and Organ Culture 84: 199–211.

[nph14849-bib-0064] Vogel J , Garvin D , Mockler T , Schmutz J , Rokhsar D , Bevan M , Barry K , Lucas S , Harmon‐Smith M , Lail K *et al* 2010 Genome sequencing and analysis of the model grass *Brachypodium distachyon* . Nature 463: 763–768. 20148030 10.1038/nature08747

[nph14849-bib-0065] Vogel J , Tuna M , Budak H , Huo N , Gu Y , Steinwand M . 2009 Development of SSR markers and analysis of diversity in Turkish populations of *Brachypodium distachyon* . BMC Plant Biology 9: 88. 19594938 10.1186/1471-2229-9-88 PMC2719641

[nph14849-bib-0066] Wang D , Amornsiripanitch N , Dong X . 2006 A genomic approach to identify regulatory nodes in the transcriptional network of systemic acquired resistance in plants. PLoS Pathogens 2: 1042–1050. 10.1371/journal.ppat.0020123 PMC1635530 17096590

[nph14849-bib-0067] Wang Y , Bouchabke‐Coussa O , Lebris P , Antelme S , Soulhat C , Gineau E , Dalmais M , Bendahmane A , Morin H , Mouille G *et al* 2015 LACCASE5 is required for lignification of the *Brachypodium distachyon* culm. Plant Physiology 168: 192–204. 25755252 10.1104/pp.114.255489 PMC4424014

[nph14849-bib-0068] Weinhold A , Bowman T . 1974 Repression of virulence in *Rhizoctonia solani* by glucose and 3‐O‐methyl glucose. Phytopathology 64: 985–990.

[nph14849-bib-0069] Yang Y , Qi M , Mei C . 2004 Endogenous salicylic acid protects rice plants from oxidative damage caused by aging as well as biotic and abiotic stress. Plant Journal 40: 909–919. 15584956 10.1111/j.1365-313X.2004.02267.x

[nph14849-bib-0070] Zheng A , Lin R , Zhang D , Qin P , Xu L , Ai P , Ding L , Wang Y , Chen Y , Liu Y *et al* 2013 The evolution and pathogenic mechanisms of the rice sheath blight pathogen. Nature Communications 4: 1424. 10.1038/ncomms2427 PMC3562461 23361014

[nph14849-bib-0071] Zhu H , Wen F , Li P , Liu X , Cao J , Jiang M , Ming F , Chu Z . 2014 Validation of a reference gene (*BdFIM*) for quantifying transgene copy numbers in *Brachypodium distachyon* by real‐time PCR. Applied Biochemistry and Biotechnology 172: 3163–3175. 24497043 10.1007/s12010-014-0742-4

