# Supplementary material for: Salicylic acid‐dependent immunity contributes to resistance against Rhizoctonia solani, a necrotrophic fungal agent of sheath blight, in rice and Brachypodium distachyon
Source: New Phytol. 2017 Oct 19;217(2):771–83. doi: 10.1111/nph.14849 (PMC5765516; doi:10.1111/nph.14849)
Supplement: Supplementary file 1 — Fig. S1 Hyphal growth of Rhizoctonia solani on nutrient agar medium containing phytohormones. Fig. S2 Effects of phytohormones on Brachypodium distachyon disease resistance to Pyricularia oryzae and Botrytis cinerea. Fig. S3 Rhizoctonia solani biomass in Brachypodium distachyon leaves at the initial infection stage. Fig. S4 Endogenous levels of phytohormones in Brachypodium distachyon accessions Bd21 and Bd3‐1 after inoculation with Rhizoctonia solani. Table S1 Primers used in this study Table S2 Summary of the sequence reads from RNA‐seq analysis mapped to the Brachypodium distachyon Bd21 genome Table S3 Differentially expressed genes (DEGs) of Brachypodium distachyon in salicylic acid treatment Table S4 Differentially expressed genes (DEGs) of Brachypodium distachyon in benzothiadiazole treatment Table S5 Cell wall biogenesis (GO:0042546)‐related genes in the differentially expressed genes (DEGs) specifically induced by salicylic acid [file NPH-217-771-s001.pdf]

New Phytologist

***New Phytologist* Supporting Information**

Article title: Salicylic acid-dependent immunity contributes to resistance against *Rhizoctonia solani*, a necrotrophic fungal agent of sheath blight, in rice and *Brachypodium distachyon*

Authors: Yusuke Kouzai, Mamiko Kimura, Megumi Watanabe, Kazuki Kusunoki, Daiki Osaka, Tomoko Suzuki, Hidenori Matsui, Mikihiro Yamamoto, Yuki Ichinose, Kazuhiro Toyoda, Takakazu Matsuura, Izumi C. Mori, Takashi Hirayama, Eiichi Miami, Yoko Nishizawa, Komaki Inoue, Yoshihiko Onda, Keiichi Mochida, and Yoshiteru Noutoshi

Article acceptance date: 13 September 2017

The following Supporting Information is available for this article:

**Fig. S1** Hyphal growth of *Rhizoctonia solani* on nutrient agar medium containing phytohormones.

**Fig. S2** Effects of phytohormones on *Brachypodium distachyon* disease resistance to *Pyricularia oryzae* and *Botrytis cinerea.*

**Fig. S3** *Rhizoctonia solani* biomass in *Brachpodium distachyon* leaves at the initial infection stage.

**Fig. S4** Endogenous levels of phytohormones in *Brachypodium distachyon* accessions Bd21 and Bd3-1 after inoculation with *Rhizoctonia solani*

**Table S1** Primers used in this study.

**Table S2** Summary of the sequence reads from RNA-seq analysis mapped to the *Brachypodium distachyon* Bd21 genome.

**Table S3** Differentialy expressed genes (DEGs) of *Brachypodium distachyon* in the salicylic acid treatment. (see separate file)

**Table S4** Differentialy expressed genes (DEGs) of *Brachypodium distachyon* in the benzothiadiazole treatment. (see separate file)

**Table S5** Cell wall biogenesis (GO:0042546) related genes in the differentially expressed genes (DEGs) specifically induced by salicylic acid.

**Fig. S1** Hyphal growth of *Rhizoctonia solani* on nutrient agar medium containing phytohormones. Mycelial agar plugs of *R. solani* AG-1 were inoculated on potato dextrose agar (PDA) medium containing 0.5 % (v/v) dimethyl sulfoxide (DMSO), 0.5 % (v/v) DMSO with 25 µg/mL hygromycin B, 1 mM salicylic acid (SA), 1 mM jasmonic acid (JA), or 1 mM ethylene (ET). Photos were taken 24 h after incubation at 25 °C. Similar results were obtained in three independent experiments.

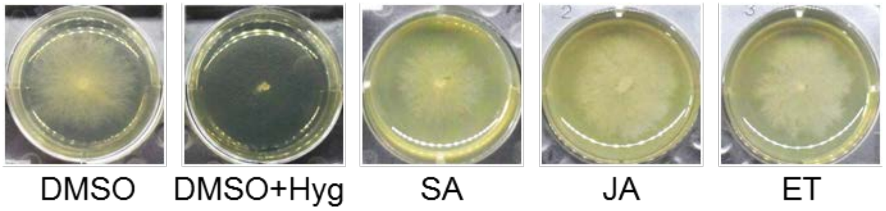


**Fig. S2** Effects of phytohormones on *Brachypodium distachyon* disease resistance to *Pyricularia oryzae* and *Botrytis cinerea.* (a, b) Lesion formation by blast fungus (a) and fungal biomass of grey mold (b) in *B. distachyon* leaves treated with water (Mock) or a 1 mM solution of salicylic acid (SA), jasmonic acid (JA), or ethylene (ET). Lesion sizes caused by *P. oryzae* were measured at 5 d post-inoculation (dpi). Results are means ± SEM, $n$ = 7; ***, $P < 0.001$ by using Student's *t*-tests to Mock. *B. cinerea* biomass (linear scale) in the inoculated leaves was measured at 3 dpi. Results are means ± SEM, $n$ = 4; *, $P < 0.05$ by using Student's-*t* tests to Mock.

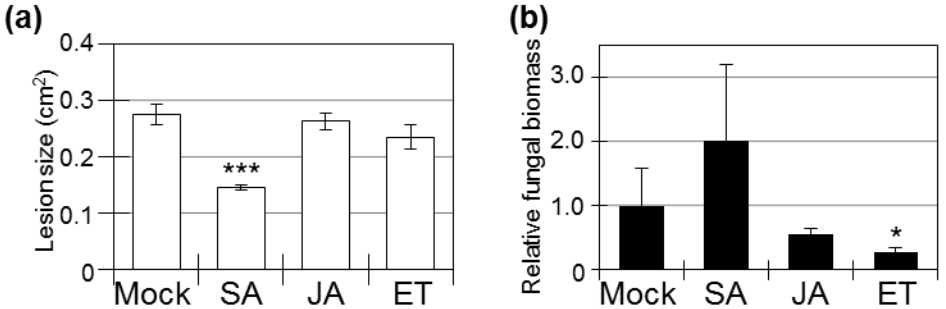

**Fig. S3** *Rhizoctonia solani* biomass in *Brachypodium distachyon* leaves at the initial infection stage. *R. solani* biomass was measured by semi-quantitative polymerase chain reaction (PCR) analysis using genomic DNAs extracted from *B. distachyon* leaf samples inoculated with *R. solani* AG-1 at 0, 5, or 20 hpi using primers for *28S rDNA* or *B. distachyon BdFIM*. Representative results obtained in two independent experiments are shown.

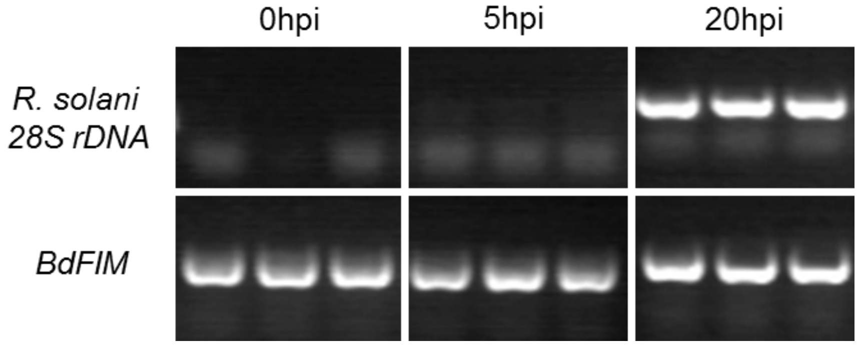

**Fig. S4** Endogenous levels of phytohormones in *Brachypodium distachyon* accessions Bd21 and Bd3-1 after inoculation with *Rhizoctonia solani.* Phytohormone levels were measured by liquid chromatography tandem-mass spectrometry (LC-MS/MS) from the leaves of *B. distachyon* Bd21 or Bd3-1 at 0 and 24 h after inoculation with *R. solani* AG-1. Results are means ± SD of three biological replicates. Results from three independent experiments are presented. SA, salicylic acid. JA, jasmonic acid. JA-Ile, jasmonic acid-isoleucine. IAA, indole acetic acid. tZ, trans-zeatin. iP, isopentenyladenine. ABA, abscisic acid.

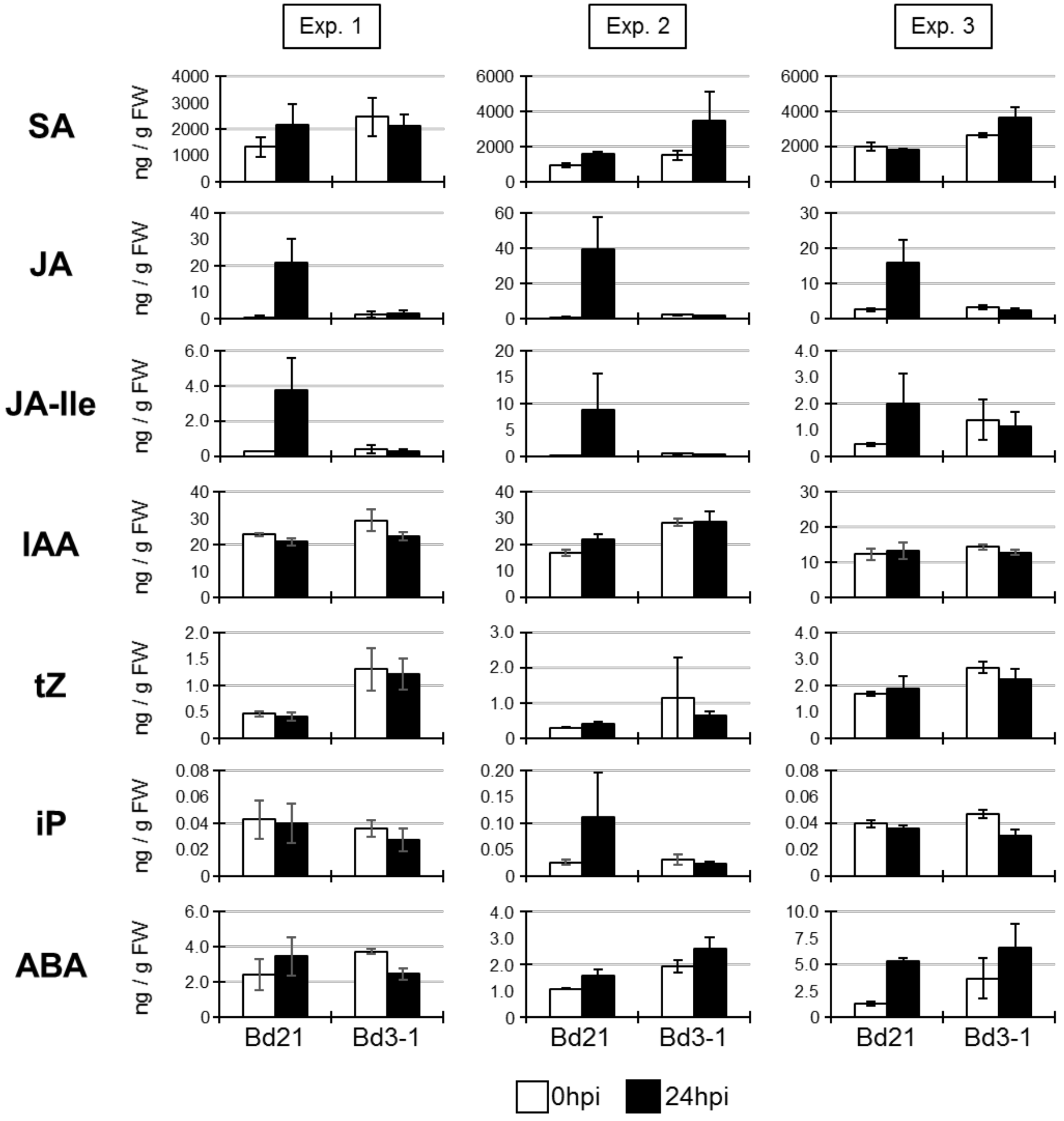

**Table S1** Primers used in this study.

| Name | Sequence | Target | References |
|---|---|---|---|
| **1) Fungal biomass measurement** | | | |
| Rs-1F | GCCTTTTCTACCTTAATTTGGCAG | *R. solani* AG-1 IA rDNA | Sayler & Yang, 2007 |
| Rs-2R | GTGTGTAAATTAAGTAGACAGCAAATG | | |
| AG5-F | TGATCAGGTGCTCGATGTCGT | *R. solani* AG-5 β-tubulin | Budge *et al.*, 2009 |
| AG5-R | CCCTGCAAGCAGTCGGTT | | |
| Bc3-F | GCTGTAATTTCAATGTGCAGAATCC | *B. cinerea* rDNA | Diguta *et al.*, 2010 |
| Bc3-R | GGAGCAACAATTAATCGCATTTC | | |
| BdFIM-F | CCTCACACGGATTTCGAGAGA | *Bradi2g13800* (*BdFIM*) | Zhu *et al.*, 2014 |
| BdFIM-R | GGACAACCCATTTCTGCGA | | |
| **2) Gene expression analysis** | | | |
| W45L1-F | GGACACCTTCAGGGTGACAT | *Bradi2g30695* (*BdWRKY45L1*) | Kouzai *et al.*, 2016 |
| W45L1-R | TTGTCGTCGTGGTAGGAGTG | | |
| W45L2-F | GATCGGAGGTGCAGAGAGAG | *Bradi2g44270* (*BdWRKY45L2*) | |
| W45L2-R | GTGTGCACCGGAAGTAGGAT | | |
| AOS-F | ACCGCCTGGACTTCTACTAC | *Bradi1g69330* (*BdAOS*) | |
| AOS-R | GAGGTTCTTCTTCTCCACCT | | |
| CESA4-F | ACAGGATCGACAAGTGGAAGAC | *Bradi3g28350* (*BdCESA4*) | |
| CESA4-R | CGTTCTTGTCATCGTCATCATC | | |
| CESA7-F | ATGAACGCTCTTATCCGTGTCT | *Bradi4g30540* (*BdCESA7*) | |
| CESA7-R | ATTGCACATAGCAGACCTTCCT | | |
| CESA8-F | GATGAGCTTCGAGAAGTCGTTT | *Bradi2g49912* (*BdCESA8*) | |
| CESA8-R | ATGGATGGCTTCTTTGATCAGT | | |
| LAC6-F | AGATGGGTTCAAGCTCAATGTT | *Bradi1g74320* (*BdLAC6*) | |
| LAC6-R | GCATCAACCTCAACCACTGTTA | | |
| LAC10-F | GCCAAACCACCAACGTACTACT | *Bradi2g54680* (*BdLAC10*) | |
| LAC10-R | GATGAACTTGGGTCTTCATGGT | | |
| Ubi4-F | TGACACCATCGACAACGTGA | *Bradi3g04730* (*Ubi4*) | Chambers *et al.*, 2012 |
| Ubi4-R | GAGGGTGGACTCCTTCTGGA | | |

**Table S2** Summary of the sequence reads from RNA-seq analysis mapped to the *Brachypodium distachyon* Bd21 genome.

| Libraries | Biological replicates | Number of checked reads | Number of mapped reads | Percentage of mapped reads |
|---|---|---|---|---|
| | 1 | 93718318 | 89150853 | 95.1 |
| DMSO | 2 | 81091570 | 77281440 | 95.3 |
| | 3 | 66906560 | 62805938 | 93.9 |
| | 1 | 75241210 | 71257417 | 94.7 |
| SA | 2 | 80393290 | 76241699 | 94.8 |
| | 3 | 86608596 | 81977749 | 94.7 |
| | 1 | 95395626 | 90101360 | 94.5 |
| BTH | 2 | 88979350 | 84447194 | 94.9 |
| | 3 | 71158628 | 66929726 | 94.1 |

**Table S5** Cell wall biogenesis (GO:0042546) related genes in the differentialy expressed genes (DEGs) specifically induced by salicylic acid.

| *Brachypodium* gene ID | Homologous genes with annotations of *Arabidopsis thaliana* | reads per million | | | LogFC (SA/DMSO) | References | |
|---|---|---|---|---|---|---|---|
| | | DMSO | BTH | SA | | | |
| *Bradi2g49912* | AT4G18780.1 \| cellulose synthase family protein | 43.46 | 55.20 | 93.94 | 1.15 | *BdCESA8* | Handakumbura *et al.*, 2013 |
| *Bradi4g30540* | AT5G17420.1 \| Cellulose synthase family protein | 26.79 | 40.37 | 65.41 | 1.32 | *BdCESA7* | |
| *Bradi3g28350* | AT5G44030.1 \| cellulose synthase A4 | 20.28 | 32.10 | 54.48 | 1.46 | *BdCESA4* | |
| *Bradi1g59880* | AT5G15630.1 \| COBRA-like extracellular glycosyl-phosphatidyl inositol-anchored protein family | 15.24 | 15.12 | 31.52 | 1.08 | | |
| *Bradi2g54680* | AT5G60020.1 \| laccase 17 | 9.04 | 12.07 | 25.79 | 1.55 | *BdLAC10* | Wang *et al.*, 2015 |
| *Bradi2g00220* | AT5G03170.1 \| FASCICLIN-like arabinogalactan-protein 11 | 7.05 | 9.97 | 19.94 | 1.53 | | |
| *Bradi1g25117* | AT3G03050.1 \| cellulose synthase-like D3 | 2.80 | 0.52 | 7.24 | 1.40 | | |
| *Bradi2g02320* | AT1G58370.1 \| glycosyl hydrolase family 10 protein / carbohydrate-binding domain-containing protein | 2.48 | 3.27 | 6.60 | 1.44 | | |
| *Bradi3g36697* | AT5G03760.1 \| Nucleotide-diphospho-sugar transferases superfamily protein | 2.33 | 3.63 | 4.99 | 1.14 | | |
| *Bradi4g44860* | AT2G38320.1 \| TRICHOME BIREFRINGENCE-LIKE 34 | 2.10 | 2.28 | 4.18 | 1.03 | | |
| *Bradi2g46197* | AT4G28500.1 \| NAC domain containing protein 73 | 1.95 | 2.28 | 4.13 | 1.12 | *SND2* | Handakumbura, 2014 |
| *Bradi1g74320* | AT2G38080.1 \| Laccase/Diphenol oxidase family protein | 1.39 | 1.93 | 3.80 | 1.48 | *BdLAC6* | Wang *et al.* 2015 |
| *Bradi4g33490* | AT2G04780.2 \| FASCICLIN-like arabinoogalactan 7 | 1.20 | 0.53 | 3.67 | 1.65 | | |
| *Bradi3g04460* | AT5G67210.1 \| Protein of unknown function (DUF579) | 1.29 | 1.30 | 3.18 | 1.33 | | |
| *Bradi4g32680* | AT1G72230.1 \| Cupredoxin superfamily protein | 1.14 | 1.16 | 2.60 | 1.22 | | |
| *Bradi4g31360* | AT2G05920.1 \| Subtilase family protein | 0.84 | 0.71 | 2.30 | 1.50 | | |
| *Bradi2g14690* | AT2G42580.1 \| tetratricopetide-repeat thioredoxin-like 3 | 0.76 | 0.86 | 1.59 | 1.09 | | |

## References


**Budge G, Shaw M, Colyer A, Pietravalle S, Boonham N. 2009.** Molecular tools to investigate *Rhizoctonia solani* distribution in soil. *Plant Pathology* **58**(6): 1071-1080.

**Chambers J, Behpouri A, Bird A, Ng C. 2012.** Evaluation of the use of the polyubiquitin genes, *Ubi4* and *Ubi10* as reference genes for expression studies in *Brachypodium distachyon*. *PLoS One* **7**(11): e49372.

**Diguta C, Rousseaux S, Weidmann S, Bretin N, Vincent B, Guilloux-Benatier M, Alexandre H. 2010.** Development of a qPCR assay for specific quantification of B*otrytis cinerea* on grapes. *FEMS Microbiology Letters* **313**(1): 81-87.

**Handakumbura P. 2014.** *Understanding the transcriptional regulation of secondary cell wall biosynthesis in the model grass Brachypodium distachyonUnderstanding the transcriptional regulation of secondary cell wall biosynthesis in the model grass Brachypodium distachyon.* PhD Thesis, Graduate School of the University of Massachusetts Amherst, Amherst, MA, USA.

**Handakumbura P, Matos D, Osmont K, Harrington M, Heo K, Kafle K, Kim S, Baskin T, Hazen S. 2013.** Perturbation of *Brachypodium distachyon CELLULOSE SYNTHASE A4* or *7* results in abnormal cell walls. *BMC Plant Biology* **13**: 131.

**Kouzai Y, Kimura M, Yamanaka Y, Watanabe M, Matsui H, Yamamoto M, Ichinose Y, Toyoda K, Onda Y, Mochida K, et al. 2016.** Expression profiling of marker genes responsive to the defence-associated phytohormones salicylic acid, jasmonic acid and ethylene in *Brachypodium distachyon*. *BMC Plant Biology* **16**: 59.

**Sayler R, Yang Y. 2007.** Detection and quantification of *Rhizoctonia solani* AG-1 IA, the rice sheath blight pathogen, in rice using real-time PCR. *Plant Disease* **91**(12): 1663-1668.

**Wang Y, Bouchabke-Coussa O, Lebris P, Antelme S, Soulhat C, Gineau E, Dalmais M, Bendahmane A, Morin H, Mouille G, et al. 2015.** LACCASE5 is required for lignification of the *Brachypodium distachyon* culm. *Plant Physiology* **168**(1): 192-204.

**Zhu H, Wen F, Li P, Liu X, Cao J, Jiang M, Ming F, Chu Z. 2014.** Validation of a reference gene (*BdFIM*) for quantifying transgene copy numbers in *Brachypodium distachyon* by real-time PCR. *Applied Biochemistry and Biotechnology* **172**(6): 3163-3175.
